# Landscape changes caused by the 2024 Noto Peninsula earthquake in Japan

**DOI:** 10.1126/sciadv.adp9193

**Published:** 2024-12-04

**Authors:** Yo Fukushima, Daisuke Ishimura, Naoya Takahashi, Yoshiya Iwasa, Luca C. Malatesta, Takayuki Takahashi, Chi-Hsien Tang, Keisuke Yoshida, Shinji Toda

**Affiliations:** ^1^ International Research Institute of Disaster Science, Tohoku University, Sendai, Japan.; ^2^ Department of Geography, Tokyo Metropolitan University, Tokyo, Japan.; ^3^ Graduate School of Science, Tohoku University, Sendai, Japan.; ^4^ Center for Education and Research of Disaster Risk Reduction and Redesign, Oita University, Oita, Japan.; ^5^ Earth Surface Process Modelling, GFZ German Research Center for Geosciences, Potsdam, Germany.

## Abstract

Landscapes are shaped by tectonic, climatic, and surface processes over geological timescales, but we rarely witness the events of marked landscape change. The moment magnitude 7.5 Noto Peninsula earthquake in central Japan was caused by a large thrust faulting, up to nearly 10 meters of slip, that expanded more than 150 kilometers along the fault zone. The deformation field reconstructed from satellite data and field surveys reveals up to 4.4 meters of uplift and associated coastal advance along the entire northern coast of the peninsula, meter-scale systematic movement of the mountain slopes consistent with slip on flexural faults, and activation of secondary inland faults, suggesting synchronized ruptures. The findings show excellent consistency between the coseismic deformation and geomorphic features and provide a vivid example of the role of a major earthquake in landscape formation.

## Teaser

Deformation due to the Noto Peninsula earthquake showed that its repetition shaped the Peninsula’s modern landscapes.

## INTRODUCTION

Landscapes that we see today are the cumulative result of various tectonic and geomorphic processes over geological timescales (*1*–*4*). In tectonically active regions, faulting can dominate landscape formation, either directly or indirectly. Earthquakes contribute to mountain building by rock uplift, and the associated shaking triggers landslides and other mass wasting processes that, in turn, affect rivers and floodplains. However, it is often unclear which role earthquakes play in landscape formation until they occur.

**Fig. 1.**
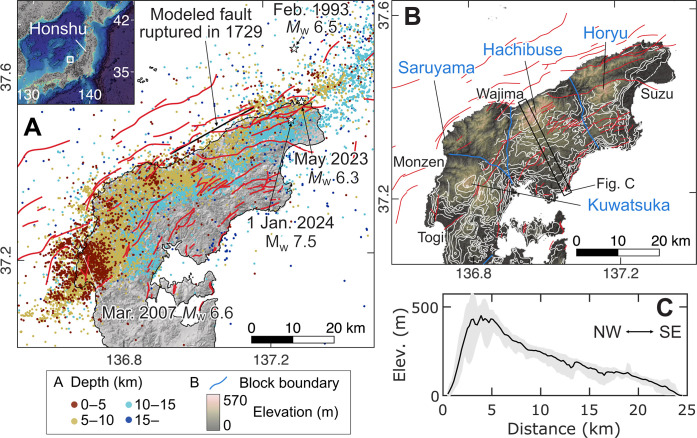
Tectonic setting of the North Noto. (**A**) Star and color-filled circles denote the epicenters of the mainshock determined by the Japan Meteorological Agency (JMA) and aftershocks relocated for this study, respectively. Red lines are mapped active faults (*57*). Locations of the 1993, 2007, and 2023 earthquake epicenters are from the JMA catalog, and the fault rupture extent for the 1729 earthquake is from (*26*). (**B**) Tectonic blocks (separated by blue lines) (*9*) and the outline of former shorelines from the Last Interglacial to ~1 million years ago at the highest elevation (white lines) (*5*). (**C**) Topographic profile across the North Noto. The elevation range in the rectangular box indicated in (B) and the average curve are shown in gray and black, respectively.

The Noto Peninsula stands as the largest peninsula along the northern coast of the Japanese mainland Honshu (Fig. 1). It has more than 10 levels of marine terraces recording ongoing uplift over the past 10^6^ years (*5*) and is composed of several low-relief (<600 m) mountain blocks. The seismotectonics of the back-arc region of the northern half of Honshu Island is characterized by reverse motion on former normal faults now under compression (*6*, *7*). The moment magnitude (*M*_w_) 7.5 Noto Peninsula earthquake occurred along one such fault, releasing the northwest (NW)–southeast (SE) compressional stress in the region (*8*, *9*). As indicated by the hypocenter distribution of the mainshock and aftershocks (Fig. 1), the fault rupture started beneath the northeastern tip of the peninsula, where an active seismic swarm and associated crustal deformation have taken place since the end of 2020 (*10*, *11*). The rupture extended more than 150 km along an offshore fault system lying parallel to the northern coastline of the peninsula. This earthquake, the largest ever recorded in the region, shook the northern area of the Noto Peninsula from extremely short distances of less than 10 km, leading to devastating damages.

The Japan Aerospace Exploration Agency (JAXA) conducted emergency observations by the ALOS-2 satellite immediately after the 1 January 2024 earthquake, which provided an exceptional dataset, enabling detailed analysis of the resulting deformation. In this study, we reconstructed a fully three-dimensional (3D) deformation field by conducting a set of synthetic aperture radar (SAR) pixel offset analyses (*12*) and applying a merging method using displacements captured by Global Navigation Satellite System (GNSS) measurements. The deformation field thus obtained reveals marked landscape changes, notably the emergence of new marine terraces and large-scale hillslope slumps.

## RESULTS

### Deformation on the peninsula and the fault rupture

**Fig. 2.**
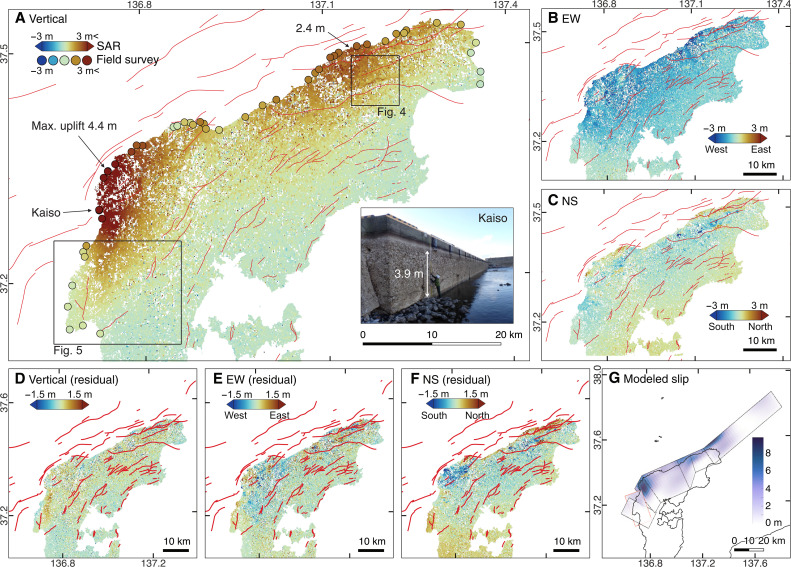
Deformation field revealed from the SAR image analysis and coastal survey. (**A**) Vertical component. Red lines are active fault traces (*57*). Filled circles are values from the field survey. Inset shows a photo at the Kaiso port. (**B**) East-west (EW) component. (**C**) North-south (NS) component. (**D** to **F**) Same as (A) to (C) except that the displacements due to the fault-slip model are subtracted. (**G**) Fault-slip model. Red outline marks the fault segment ruptured during the 2007 earthquake.

The 3D deformation field was reconstructed using the displacements in the line-of-sight and horizontal (parallel to satellite track) directions obtained from each of the seven SAR image pairs (Materials and Methods). The obtained deformation field is characterized by (i) prominent uplift along the northern coast of the peninsula in the vertical component with a global maximum of 4.4 m on the western edge and a secondary local maximum of 2.4 m in the northeast around longitude 137.1°E, (ii) up to ~2 m of westward movement of the northern part of the peninsula, and (iii) undulating horizontal displacements of ~1.5 m distinctly identified in the north-south (NS) component (Fig. 2). The first two kinds of deformation mainly result from the oblique thrust motion of the main fault rupture. The undulating signal fits downslope displacement across inland valleys.

Photo credit: Daisuke Ishimura, Tokyo Metropolitan University

We confirmed that the signals obtained in the mountain ranges are not artifacts due to the stereoscopic effect of the difference in the satellite locations (Materials and Methods). On the mountain ranges, numerous landslides of less than a few hundred meters have been identified from photographic surveys (*13*, *14*), but most of them were masked out in our results in the filtering process. Therefore, the horizontal displacements (Fig. 2, B and C) mainly contain the displacements due to the fault rupture and range-wide hillslope slumps.

To separate the deformation due to fault slip from other geomorphic changes, we estimated a fault-slip model by inverting the displacements obtained from the SAR image analysis and those captured by the national GNSS Earth Observation Network System (GEONET; Materials and Methods). We estimated slip distribution on three curved fault planes: (i) a 150-km-long fault along the northern coast similar to the one assumed by the national tsunami hazard evaluation (*15*), (ii) a segment striking east-northeast–west-southwest (WSW) (Monzen-oki segment) that caused the 2007 *M*_w_ 6.6 Noto Peninsula earthquake on the western side (*16*–*22*), and (iii) the last segment striking north-northeast (NNE)–south-southwest (SSW) located south of the Monzen-oki segment (Fig. 2G and Materials and Methods). The last southwestern segment is assumed on the basis of our strong motion data analysis that identified an NS-striking thrust-type sub-event (Materials and Methods, fig. S13) and the presence of NS-striking anticline structures around this area and more to the south (*23*). The fault exhibits an along-strike variation in the dip angle, ranging from ~60° east of the mainshock to ~45° west of the mainshock, as suggested by relocated aftershock distribution (fig. S1).

Up to ~10 m of slip was estimated along the coast of the Noto Peninsula, with two particularly large slip patches near the two uplift areas (Fig. 2, A and G, and fig. S9). The eastern large-slip patch has both dip-slip and right-lateral strike-slip components, resulting in oblique motion. The western large-slip patch along the segment striking more to the NS direction is predominantly dip slip. In summary, the fault-slip inversion indicates that the westward motion of the peninsula was backstopped by the fault structure to the west, creating prominent uplift up to 4.4 m at the western edge and releasing the NW-SE directing compressional stress that had been accumulating in the North Noto (*8*). The moment release estimated by our slip model is equivalent to *M*_w_ 7.5, broadly consistent with the one determined by seismological methods (table S4) (*24*). It is worth noting that our model shows little slip on the Monzen-oki segment that ruptured during the 2007 earthquake (red box in Fig. 2G; see also fig. S9). This indicates that the Monzen-oki segment acted as a barrier to hinder further rupture propagation to the south.

### Uplift and formation of new marine terraces

On the North Noto, Pleistocene marine terraces are widely distributed across the Peninsula except for its northern flank (Fig. 1B). Only Holocene and rare Last Interglacial [125 thousand years ago (ka)] terraces exist along the northern coast and their elevation reflects a strong tilting to the south (*5*, *25*). The analysis of both geological structures and marine terraces ≤125 ka indicates an SSW tilting of the Peninsula with a maximum uplift rate of 0.8 to 1.0 mm/year in North Noto (*5*, *25*), which is consistent with the gradually decreasing elevation from north to south (Fig. 1C). The distribution of greater coseismic uplift in the north from the SAR image analysis (Fig. 2A) strongly suggests that the repeated occurrence of 2024-type earthquakes is a main driver of crustal deformation across the Noto Peninsula over the past hundred thousand years.

**Fig. 3.**
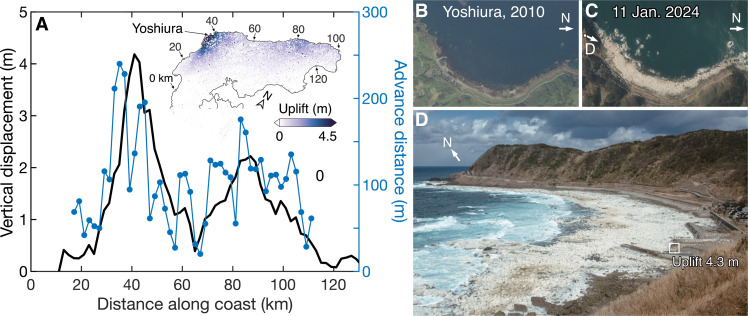
Uplift and coastal advance along the coast. (**A**) Uplift (black) and coastal advance distance (blue) taken along the shoreline (shown in the inset). (**B** and **C**) Orthorectified aerial images taken in 2010 and 2024, respectively (*14*). (**D**) The bedrock and boulder platform emerged due to coastal uplift.

The coseismic displacement uplifted large swaths of bedrock and sediment out of the intertidal and subtidal zones. The coastline accordingly advanced by up to 200 m in the NW (Fig. 3 and Materials and Methods). The emerged area forms extensive raised benches and sloping platforms. They will be progressively eroded by waves, but the widest ones will most likely be preserved as marine terraces in the geomorphic record. Past earthquakes smaller than *M*_w_ 7.0 also contribute to the uplift of the Peninsula, but it is unlikely that they raise platforms, if any, large enough to be preserved (*26*–*28*).

Photo credit: [(B) and (C)] Geospatial Information Authority of Japan (GSI) and (D) Luca C. Malatesta, GFZ German Research Center for Geosciences

On the basis of the comparison of SAR and optical images acquired before and after the earthquake, we find that the length of shoreline advance is roughly proportional to the amount of coastal uplift with an approximate ratio of 60, implying that the average near-shore slope of the platform is ~1.7% (Fig. 3A). The total newly emerged area was estimated to be 4.5 km^2^.

### Hillslope slump

**Fig. 4.**
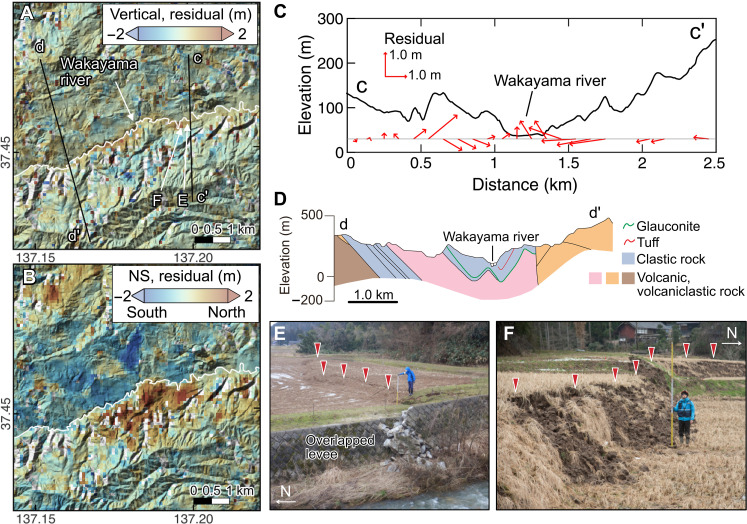
Displacements associated with the hillslope slump around the Wakayama River. (**A** and **B**) Vertical and NS displacements after removing the contribution from the fault slip. (**C**) Elevation profile along the c-c′ line in (A) (black) and the displacements along the same line. The arrows show the vertical and horizontal (parallel to the profile line) components derived from the displacement field after correcting for the contribution from the fault slip. Elevation is based on a 10-m meshed digital elevation model obtained from GSI. (**D**) Geological cross section along the d-d′ line in (A) from (*66*). (**E** and **F**) Photos of the vertical and contractional displacements found close to the Wakayama River.

Widespread surface displacement was identified in the Horyu and Saruyama mountain ranges (Figs. 1 and 2). As a general tendency, we can identify hillslopes being displaced downslope (Fig. 4B), making ridges and valleys divergent and convergent boundaries, respectively. In these areas, numerous landslides and surface failures of smaller scales were identified in aerial photographs (*14*), most of which were masked out in our results in the filtering process described earlier.

Photo credit: (E) Takayuki Takahashi, International Research Institute of Disaster Science, Tohoku University, and (F) Luca C. Malatesta, GFZ German Research Center for Geosciences

Along the Wakayama River in particular, localized convergence is clearly visible in the horizontal component (Fig. 4, Materials and Methods, and fig. S18). Displacement toward the valley was observed over a wide area (~5 km) on both valley flanks, although no clear large-scale landslides were identified in the aerial photographs. These observations suggest that range-wide coseismic hillslope slumps may occur on preexisting weak surfaces beneath both sides of the Wakayama River.

The localized convergence in the valley is accompanied by an uplift of up to 2 m in a narrow zone parallel to the thalweg. We conducted a field survey to record the amount and sense of displacement of the surface deformations (e.g., scarp and warping) that appeared along the Wakayama River. We confirmed that the geomorphic features of the local uplift and accompanied contraction partially occurred along existing scarps and are consistent with a compressional ridge caused by the slopes sliding from both valley flanks (Fig. 4, E and F; Materials and Methods; and figs. S21 and S22).

Moreover, aerial photos reveal extensional cracks along a ridge north of the Wakayama River (fig. S23). The spot is characterized by a double ridgeline, suggesting repeated extensional events. The distribution of the cracks is consistent with the divergent NS displacements, providing key evidence for the occurrence of coseismic slumping.

The geological cross section close to the area of the compressional ridge along the Wakayama River shows that the siliceous siltstone unit of the Iizuka Formation is separated from an older volcaniclastic unit by a thin layer of glauconite sandstone at a depth of 200 to 300 m (Fig. 4, Materials and Methods, and fig. S19). Glauconite is commonly associated with landslides and can form a plane of weakness (*29*–*33*). Here, the Wakayama River sits in a broad syncline and the glauconite layer dips parallel to the valley flanks. Below the valley floor, a secondary fold in the glauconite layer is consistent with meter-scale deformation observed at the surface (Fig. 4F, Materials and Methods, and fig. S19). On the basis of these observations, we infer that the glauconite layer is a likely slipping plane for the slump. More precisely, the slumps can be described as rotational slides in weak rock with a long, and close to planar, uphill section (*34*). The localized compression caused by the convergence of both slumps would have then resulted in the pronounced surface deformation of the valley floor.

### Inland synchronized faulting

**Fig. 5.**
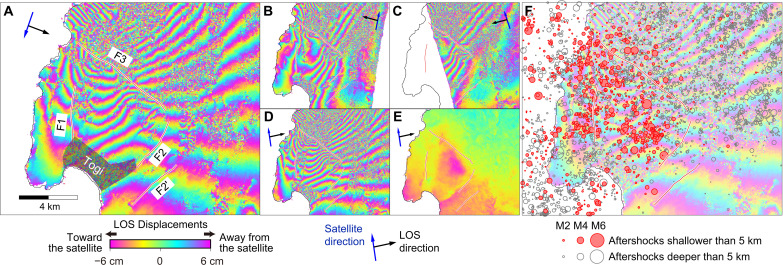
Synchronized faulting in the Kuwatsuka block. (**A** to **D**) Coseismic SAR interferograms [(A) Path 26, (B) Path 20, (C) Path 121, and (D) Path 127]. The signal in the Togi District is decorrelated and masked with gray shade in (A). (**E**) Postseismic SAR interferogram computed from the images acquired on 3 and 17 January 2024 from the orbit Path 127. One color cycle corresponds to 12 cm of line-of-sight (LOS) displacements. The red lines F1, F2, F2′, and F3 denote major displacement discontinuities identified in the interferograms. (**F**) Aftershock distributions superimposed on the interferogram shown in (A).

The Kuwatsuka block in the southwestern part of the North Noto (Fig. 1B) is characterized by numerous centimeter-level displacement discontinuities as shown in the SAR interferograms (Fig. 5). Some of the displacement discontinuities are located close to previously mapped faults (*9*). The interferograms are decorrelated on the alluvial lowland in the Togi District (shaded area on Fig. 5A), which prevents us from tracking the discontinuity traces in its surrounding area. The identified displacement discontinuities are interpreted as “synchronized” fault ruptures being activated during the mainshock. Such secondary fault ruptures have often been observed after shallow crustal earthquakes such as the 2016 Kumamoto earthquake (*35*, *36*), the 2019 Ridgecrest earthquake (*37*), and the 2021 Haiti earthquake (*38*). This Kuwatsuka block area is characterized by intense shallow aftershocks (Figs. 1A and 5F). The aftershock-prone zone is bounded by the main rupture fault off-shore to the west and the fault east of the Togi District (F2 in Fig. 5). These suggest that the fault F2 acts as a structural boundary partitioning the weaker rock unit to the west and the stronger unit to the east.

An interferogram captured after the 2024 earthquake shows a rapid postseismic uplift or westward displacement in the triangular area confined by the faults F1, F2, and F3 (Fig. 5E). From the spatial distribution of the Pleistocene marine terraces, it is inferred that the Noto Peninsula is divided into small blocks, each showing southward tilting (*5*). The observed coseismic and postseismic deformation within the Kuwatsuka block is consistent with such geomorphic observation.

From the present study, it is not clear whether the inland faults ruptured passively in an aseismic manner, ruptured seismically with the main rupture, or ruptured seismically during the aftershock sequence. This highlights an important question for earthquake hazard evaluation, that is, whether these faults are capable of rupturing spontaneously to generate earthquakes or whether they are only activated passively by the stress changes due to other large earthquakes.

## DISCUSSION

Before the occurrence of the 2024 earthquake, it was known that the Noto Peninsula had been uplifting over the last million years and that slip on reverse faults could explain the uplift. Still, little evidence constrained past fault activity and its role building the landscape. The striking landscape changes due to the January 2024 earthquake revealed by the SAR image analyses and field survey, i.e., the uplift pattern along the coast, hillslope slumps, and secondary inland faulting, is a remarkable match for the local geomorphic and geological features. This strongly suggests that the repeated occurrence of earthquakes of the same kind is a dominant driver of landscape building in the North Noto recorded as marine terraces of the Last Interglacial (125 ka) and younger periods.

Although direct evidence of past earthquakes of the same kind has not been found, their proposed repetition is supported by the facts that the Noto Peninsula is in the seismic belt of the Japan Sea eastern margin, where several large earthquakes such as the 1983 Central Japan Sea earthquake (*M*_w_ 7.7) and the 1964 Niigata earthquake (*M*_w_ 7.6) have occurred and that a dense network of active faults is identified offshore. This is consistent with a report published in August 2024 by the Earthquake Research Committee of the Headquarters of Earthquake Research Promotion of Japan, which evaluated that the maximum size of the earthquakes along the fault zone that ruptured in January 2024 can be *M*_w_ 7.8 to 8.1 (*39*). Three levels of Holocene marine terraces along the northern coast (*25*) may also provide a record of previous events (*28*) although the extent of the potential associated coseismic displacement remains to be quantified.

The hillslope slumps captured in this study have a spatial extent of more than a few kilometers and the one along the northern coast near the northeastern edge of the peninsula was as long as ~15 km (figs. S5 and S18). It is the first time that such large-scale slumps and their associated toe scarps are identified and quantified with satellite data. With the progress in measurement technology, we are and will be able to capture subtle and broadly distributed signals such as those identified in this study. These coseismic surface processes, which have been neglected in the past, may play important roles in landscape building. Our approach allows a greater scrutiny of the distributed surface processes accompanying large earthquakes. It opens opportunities to study landscape building in seismically active regions.

## MATERIALS AND METHODS

### Aftershock distribution analysis

We relocated aftershock hypocenters in the study region from 1 to 31 January 2024, using the double-difference relocation method (*40*). Our relocation covered 17,202 earthquakes with *M*_JMA_ ≥ 1 listed in the Japan Meteorological Agency (JMA) unified catalog, where *M*_JMA_ represents the local magnitude scale used by the JMA. The relocation process is the same as that of a previous study (*41*) with the 1D velocity model of the JMA (*42*). We used 481,997 *P*-wave and 415,806 *S*-wave differential arrival time data from the arrival time data in the JMA unified catalog. We also used 3.78 million *P*-wave and 3.82 million *S*-wave differential arrival time data derived from the waveform correlation analysis. Waveform data were obtained from 28 local and regional stations of the National Institute for Earth Science and Disaster Prevention (NIED) Hi-net (*43*), national universities, and the JMA.

The relocated aftershocks shown in fig. S1 are concentrated on a relatively simple south-SE dipping plane structure from near the mainshock hypocenter to the west, likely representing the mainshock rupture planes. However, the aftershock distribution becomes quite complicated near the western end. The western edge is very close to the 2007 *M*_w_ 6.7 earthquake fault, but most of the aftershocks have occurred east of it. The model faults obtained in this study (see the “Fault-slip inversion using deformation data” section) are broadly consistent with the aftershock distribution (fig. S1), although a systematic difference in the dip angle of the fault model and the aftershock distribution is observed on the eastern part of the peninsula. Most of the slip on this segment is confined to depths shallower than the aftershock depths (fig. S9), which may be a topic of investigation in future studies.

### Deformation analysis using SAR images

#### Data and processing

In this study, seven pairs of strip map–mode ALOS-2 SAR images were used (table S1). For each pair, interferometric SAR (*44*) and pixel offset analysis (*12*) were conducted using the RINC software (*45*) and alos2App of the ISCE software (*46*), respectively (fig. S2). The SAR pixel offset analysis results were used for constructing the 3D displacement field. We assumed that all the measured displacements were caused coseismically, neglecting the effects of the displacements before and after the occurrence of the *M*_w_ 7.5 earthquake. This is justified by the fact that our target is meter-scale geomorphic deformation.

A pixel offset analysis on a pair of SAR images outputs displacements in the range and azimuth components. The range component records the displacements in the line-of-sight direction, i.e., the distance change between the radar antenna and the ground. The azimuth component records the horizontal displacements parallel to the satellite’s flight direction. We used overlapping windows of 64 by 64 pixels, approximately corresponding to 90 m by 140 m in the range and azimuth directions, for calculating the correlation and thus the offsets in the two radar images. We also masked low-correlation values and then applied median filters with a 3-by-3 pixel window. As a result, signals from landslides having scales less than a few hundred meters are masked out.

#### Sources of errors

The main sources of errors in pixel offset analysis are the decorrelation (*12*, *47*), atmospheric delay noise (*48*–*50*), stereoscopic effect (*12*, *51*), systematic bias caused by the image-wide deformation, and the preseismic and postseismic displacements. The error from the decorrelation was mitigated by the masking and median filtering mentioned above. The atmospheric delay noise in the range component is less than or equal to around 10 cm and can be neglected. The atmospheric delay noise in the azimuth component can be as large as a few meters because of the ionospheric disturbance (*48*); this type of noise commonly has long wavelengths and can be corrected by referencing with the GNSS displacements (see the “Reconstruction of the 3D displacement field” section).

The stereoscopic effect appears in such a way that the artifact is correlated with altitude when the digital elevation model used for correction is not accurate enough. This effect is amplified when the ground elevation is high and the perpendicular baseline is large, which is not the case for this study (maximum perpendicular baseline was 263 m). We further confirmed that this effect is negligible (less than ~10 cm) by conducting the pixel offset analysis on pairs of preseismic SAR images (fig. S2).

The basic principle of the pixel offset analysis is that the deformation existing in a pair of co-registered images corresponds to the real deformation of the Earth’s surface. Thus, if the deformed area is relatively large compared to the area used to co-register the pair of images, then the real deformation of the Earth’s surface is “absorbed” in the co-registration, which causes systematic bias. In this study, this systematic bias is corrected by referencing the GNSS displacements.

The preseismic displacements (displacements between the first SAR acquisition and just before the occurrence of the earthquake) included in the SAR pixel offset results are considered to be 20 cm or less. This is because the most prominent deformation during the seismic swarm period was due to the 5 May 2023 *M*_w_ 6.2 earthquake, causing up to 20 cm of uplift along the northern coast at the northeastern tip of the peninsula (*52*). The postseismic displacements have been confirmed to be in the order of several centimeters from the GNSS displacement time-series and postseismic SAR interferograms (fig. S2).

#### Reconstruction of the 3D displacement field

We used the GNSS displacements to correct the long-wavelength noise and to reference the displacement field. We used the daily coordinate solutions (F5 solutions) of the GEONET of the Geospatial Information Authority of Japan (GSI) (*53*). To obtain the coseismic displacements (fig. S3), we (i) converted the daily positions of the GEONET stations into local eastward, northward, and upward displacements; (ii) referenced them to a far-field station (station Misumi located at 34.77°N, 131.92°E); (iii) calculated the 3-day average of the displacements before (29 to 31 December 2023) and after (2 to 4 January 2024) the earthquake; and (iv) subtracted the former from the latter.

We constructed an accurate displacement field in North Noto (north of latitude 37.1°N). The procedure for removing the long-wavelength noise and referencing the displacements for each of the range or azimuth displacement data from the SAR pixel offset analysis was as follows:

1) the GNSS stations within the SAR image scene and those located to the north of latitude 37.15°N were selected;

2) the coseismic displacements of the selected GNSS stations were converted to the range or azimuth component ($u_{\text{GNSS}}$);

3) the SAR range or azimuth displacements at the GNSS station locations were extracted by taking the average of the values in a 50-by-50 pixel window located around the GNSS station location ($u_{\text{SAR}}$);

4) a constant offset *l* is estimated in such a way that the root mean square (RMS) of the differences between the SAR-based (range or azimuth displacements) and GNSS displacements in the same direction is minimized, where the RMS is defined as RMS $=\sqrt{\Sigma {\left({u^{i}}_{\text{SAR}}+l-{u^{i}}_{\text{GNSS}}\right)}^{2}/N}$, and *N* is the number of stations and *i* = 1, 2,…, *N*.

As an exception, the offset value for the azimuth displacement data from Path 26 was manually adjusted by 10 cm because the displacement values were systematically misaligned with other datasets. Also as an exception, the range and azimuth displacement data from Path 20 were referenced to the displacement field obtained using the rest of the datasets because the Path 20 scene covered only two GNSS stations.

The RMS values after referencing are listed in table S1. The range and azimuth data that had RMS values less than 16.8 and 22.6 cm, respectively, were used. These thresholds were selected to ensure the highest accuracy while retaining sufficient data from various directions to construct a 3D displacement field. This ensures that the accuracy of the constructed displacement field, excluding the contribution from the random uncorrelated noise, is within ~20 cm. The displacement data for Paths 19 and 121 contain the displacements due to the 5 May 2023 *M*_w_ 6.2 earthquake that occurred around the northeastern tip of the peninsula, but its effect is minor because its maximum uplift was ~20 cm (*52*).

Last, the referenced and retained range and azimuth displacement datasets were inverted at each pixel to derive the 3D displacements using a linear least-squares method (*54*). The number of data (displacements from different directions) is different for different pixels (fig. S4), but, in most of the areas, it is more than or equal to six. The obtained 3D displacements are shown in Fig. 2. We checked that the displacement field did not contain distinct discontinuities across the boundaries of the scenes.

To check the consistency, we also conducted a pixel offset analysis using a pair of Sentinel-2 optical images and the COSI-Corr software (fig. S5) (*55*, *56*). Although the obtained displacements are coarser in resolution and contain larger randomly decorrelated errors, the signals on the hillslopes are consistently recognized, validating the results of the SAR pixel offset analyses.

### Fault-slip inversion using deformation data

We relied on the mapped active reverse faults offshore the Noto Peninsula (*15*, *57*), the centroid moment tensor of the mainshock determined by the JMA, and the 30-day relocated aftershocks (see the “Aftershock distribution analysis” section) to reconstruct the geometry of the ruptured fault. The adopted fault geometry consists of three segments. The first segment is a 120-km-long, NE-SW–trending fault subparallel to the coastline of the peninsula. The fault exhibits an along-strike variation in the dip angle, ranging from ~60° to the east of the mainshock to ~45° to the west of the mainshock, as suggested by the hypocenter of the mainshock and the relocated aftershocks. The second segment is the seismogenic fault that ruptured during the 2007 Noto Peninsula earthquake. Last, the third segment is a NNE-SSW–trending fault located further south of the second segment, along an offshore active reverse fault cutting through the Pleistocene deposits (*23*, *57*). Given the limited constraint on the dip angle of the second and third segments from relocated aftershocks or previous studies (*18*–*22*), we opted to assign a dip angle of 45°, which was extrapolated from the nearest part of the first segment. As a result, the aftershock locations coincide with the fault at a depth of 12 km for the first segment. While we recognized the possibility of listric fault geometry, we simplified the inverse problem by assuming that the dip angle of the fault remains constant versus depth. The bottom of the fault is at a depth of 20 km. We discretized the fault planes into a series of quadrilateral subfaults, each ~2 km by 1.75 km in size, resulting in a total of 1050 subfaults.

We performed a joint inversion of GNSS data and SAR pixel offset data to constrain the complex fault kinematics during the earthquake. GNSS data offered a sparse but expansive coverage of data points, crucial for interrogating coseismic slip at depth, whereas SAR pixel offsets complemented this by providing dense data coverage on the Noto Peninsula to enhance the near-field constraint. Although SAR pixel offsets provided displacement in both range and azimuth components, we only adopted range offsets in the inversion due to a relatively large noise in the azimuth component. We excluded two GEONET GNSS stations that were probably affected by localized deformation from the inversion (stations 0575 and 0576; fig. S3). The matrix representation of the inverse problem can be formulated as

$$\left[\begin{array}{c} d^{\text{gnss}}\\ d^{\text{rgo}} \end{array}\right]=\left[\begin{array}{cc} G_{\text{slip}}^{\text{gnss}} & 0\\ G_{\text{slip}}^{\text{rgo}} & G_{\text{ramp}}^{\text{rgo}} \end{array}\right]\left[\begin{array}{c} m_{\text{slip}}\\ m_{\text{ramp}} \end{array}\right]$$

where $d^{\text{gnss}}$ is a vector composed of 3D coseismic displacements measured at each GNSS site, and $d^{\text{rgo}}$ is a vector consisting of downsampled range offsets in the descending Paths 19, 20, and 26 and ascending Paths 121, 126, and 128. $G_{\text{slip}}^{\text{gnss}}$ and $G_{\text{slip}}^{\text{rgo}}$ are Green’s functions that relate the surface displacements observed by GNSS and range offsets, respectively, to the subsurface fault slip, $m_{\text{slip}}$, in both strike-slip and dip-slip components under the homogeneous elastic half-space assumption (*58*). We adopt a shear modulus of 30 GPa and a Poisson ratio of 0.25, which are commonly used values to describe the elastic properties of the crust. In addition, we incorporate bilinear ramps for pixel offsets of each Path in the inversion to reduce long-wavelength, non-tectonic errors that may remain in the data even after the preprocessing. $m_{\text{ramp}}$ are the parameters describing bilinear ramps in the pixel offsets of each track. It is related to $d^{\text{rgo}}$ via $G_{\text{ramp}}^{\text{rgo}}$, a matrix comprising *xy* coordinates of data points of each track in a local Cartesian frame. We assign SDs of 1 and 2 cm to weight the horizontal and vertical components of GNSS data, respectively, while assigning an SD of 15 cm to weight the pixel offsets. These values agree well with the RMS levels of the differences between the model and individual datasets, as shown in table S2.

Given the right-lateral and thrust kinematics observed during the earthquake, we treat our inversion as a nonnegative least-squares problem to sample the best-fit model subject to positive right-lateral and thrust slip. We stabilize the inverted coseismic slip distribution by introducing a second-order Tikhonov regularization, given by

$$0=\lambda \;L_{\text{slip}}m_{\text{slip}}$$

where $L_{\text{slip}}$ is a discrete second-order Laplacian operator acting in both strike and dip directions. The hyperparameter λ denotes the weight of the regularization. Note that the regularization of slip on each fault is independent, while they share a unified weight. We adopt λ = 1 to balance the trade-off between the model misfit and roughness (fig. S6). We document the RMS error and variance reduction for each dataset in table S2. Model fits to GNSS and range offsets are illustrated in figs. S7 and S8, respectively. The resulting coseismic slip model is illustrated in fig. S9.

To show the reliability of the slip model, we performed checkerboard tests with the same observation data points for two different input patterns. In the first test, we used coarser (6-by-5 fault elements, ~12 km by 9 km) input checkers (fig. S10), and, in the second test, we used finer (3-by-3 fault elements, ~6 km by 5 km) input checkers (fig. S11). The first test showed that the data could recover the given slip pattern in the middle section of the fault, the 2007 Monzen-oki segment, and the southwestern-most segment, but with poor resolution for the northeast offshore segment (fig. S10). The resolution of fault slip decreased with depth. When we adopted the finer input checkers, the overall recovery of the slip pattern became worse, while the resolution of the shallowest parts remained acceptable (fig. S11). In addition, we estimated the errors in fault slip using the model covariance calculated from the data covariance and error propagation (fig. S12). The estimated SD shows along-strike variation, ranging from 0.25 to 1.7 m. The SD on the northeastern offshore fault segment is relatively large due to poor data constraints. The region where we imaged notable coseismic slip has a relatively low SD. This result ensures the reliability of our inverted slip distribution.

We also examined the model performance by assigning uniform dip angles of 40°, 45°, and 50° and a listric geometry with dip angles of 60° from 0- to 10-km depth and 30° from 10- to 20-km depth. It turns out that the above models provide variance reductions lower than the preferred model with dip varying along strike, which validates the adopted fault geometry. The variance reduction of each model is documented in table S3.

The tensorial sum of coseismic slip from our preferred model was also calculated and compared with focal mechanism solutions determined by seismic data (table S4). The result shows that the nodal planes estimated by our geodetic slip model differ slightly from those of seismic moment tensors, probably due to different datasets, but are still broadly consistent.

### Strong motion data analysis

We performed a sub-event analysis of the mainshock (*59*, *60*) by joint inversion of near-source strong-motion data and GNSS data. The method accounts for observed data by placing multiple moment tensors on the spatiotemporal grid nodes (*59*). The strong-motion data used were from NIED KiK-net (*61*), and the GNSS data were from GEONET, each with three components. From the KiK-net stations, we selected four near-source stations where the waveforms were less affected by the complexity of the real velocity structure. From the GEONET stations, we selected 63 stations surrounding the source region. Both dynamic and static Green’s functions were calculated by a method (*62*) based on the 1D structure of the JMA (*42*). The locations where point sources (sub-events) could be placed were limited to a plane on which the mainshock hypocenter is located with a strike angle of 50° and a dip angle of 40°. On the basis of the aftershock distribution, the fault model is 100 km long and 40 km wide, with 35 grid nodes equally spaced in each direction. According to the JMA unified catalog, the origin time of the mainshock is 7:10:22.4 (UTC) on 1 January, but a smaller slip began earlier at 7:10:09.54. The JMA catalog distinguishes the precursory slip as an M5.9 event from the mainshock, but the validity of this distinction is not clear. In our analysis, the time at which point sources could be placed was set to be between 7:10:09.54 and 120 s thereafter. The analyzed frequency range was 0.02 to 0.10 Hz, and the original acceleration waveforms were downsampled with a sampling interval of 0.5 s. The weights of the strong-motion and GNSS data were determined by trial and error and lastly set to 1:1, which resulted in explaining both types of data equally well. Last, the combination of the six sub-events allowed for global modeling of the mainshock observation data with a variance reduction of 75.0% for strong-motion data and 75.2% for GNSS data (fig. S13). Table S5 shows the obtained source parameters of the sub-events.

Most moment tensors obtained were of reverse fault type with WSW strike, consistent with the aftershock distribution. Two major ones are located to the east and west of the hypocenter, the latter being close to where the *M*_w_ 6.6 to 7.0 earthquake occurred in 1729. The sum of the sub-events yields a reverse fault type moment tensor with a NE-SW strike and a seismic moment of *M*_w_ 7.56, which is in good agreement with long-period solutions, including the GCMT solution. At the west end, the largest sub-event with a near N-S strike focal mechanism was obtained, which explained some of the WSW-oriented surface displacements.

### Coastal uplift survey

We used field evidence of coastal uplift to provide a ground truth to the satellite-based measurement. The survey was carried out during two field missions in January to February and early March on the Noto Peninsula, measuring a total of 52 sites (data S8 and fig. S14). The tidal range in the Noto peninsula is less than 0.5 m (*63*), and, with tidal corrections, the coseismic coastal uplift can be measured accurately by taking the difference of the sea levels before and after the earthquake.

After the earthquake, the coastline of North Noto is marked by a nearly continuous white band painting the rocks that were uplifted out of the sea (fig. S15, A and B). The white color stems from the fronds of the calcareous red seaweed, known as pirihiba in Japan, which turns from dark pink to white after death (fig. S15, C and D). Pirihiba red algae are low intertidal and subtidal species (*64*). The upper limit of the white pirihiba red algae is then a reliable proxy for the former mid- to low-tide datum on the uplifted coastal rocks. Survey of live pirihiba red algae in Kamiozawamachi confirmed their highest extent at 0.2 m below mean sea level. Besides the calcareous red algae, oysters are another sessile organism offering a reliable paleo-sea level proxy. Oysters can live in the middle to upper intertidal range and when both pirihiba red algae and oysters were found at the same site, the upper limit of the latter tended to be higher by around 0.2 m. Oysters were more commonly found on harbor walls than on exposed boulders and cliffs while pirihiba red algae were ubiquitous.

We used two survey methods to ground-truth surface uplift with field observations of the uplifted sessile organisms: direct measurement with a survey staff and distant measurement with a laser range finder.

For survey staff measurements, we targeted sites with near vertical faces that are, if possible, sheltered from the open sea, ideally harbors when they were not entirely raised out of the water by the earthquake. The base of the staff was set at the mean elevation between wave crests and troughs. The height difference with the top of pirihiba red algae and/or oysters was then recorded (fig. S15, E and F). The time of the survey was recorded to correct for the tidal level. The height of the waves at the survey points was noted to inform an error margin.

The laser range finder measurement was used when there was no accessible face to measure both water and sessile organisms with the survey staff. We used a TruPulse 200. The upper limit of pirihiba red algae or of oysters was used as a former sea-level indicator. We targeted the transition from alive to dead pirihiba red algae to constrain the current sea level. We used the average value of five repeated laser measurements each for lower and upper limits (except for site ID #7 and #8). This measurement does not need tidal correction. We targeted a single inaccessible site from the shoreline, or, maintaining a fixed position, we targeted the upper and lower constraints at different sites (e.g., a dry harbor wall and the base of an uplifted bench). We confirmed that the same values were obtained by the survey staff measurement and the laser range finder measurement at the Kaiso Port.

The precision and margins of errors of the measurements are defined as follows. Using the survey staff, we used a 10-cm precision in the field by default to reflect the variability in the position of sessile organisms. The height of waves also adds an error to the survey staff method with either 10-cm uncertainty for calm water or 20 cm for agitated water. For the laser range finder survey, we considered a 10-cm uncertainty for each of the lower and upper limits (20 cm total).

We corrected the tidal level using the theoretical tide level tables (*63*). Then, we calculated the amount of uplift from the mean sea level of +0.25 m from T.P. (Tokyo Peil) at Wajima.

We confirmed by the field survey that coastal uplift occurred in the northern part of the Noto Peninsula (fig. S14B). The uplift distribution including the two peaks of 4.3 m at Yoshiura Port and ~2 m at Otani Port and a trough of 0.2 m at Uniu Port is consistent with that derived from the SAR pixel offset analysis.

### Coastal advance analysis

We mapped newly emerged areas due to coseismic uplift based on the difference in the ALOS-2 SAR intensity images before and after the mainshock. We used five pairs of SAR images provided by JAXA, three on the ascending track, and the other two on the descending track (Paths 19, 26, 121, 126, and 127; table S1) to avoid misinterpretation associated with layover and radar shadow effects (fig. S16). The emerged rocky coast usually exhibited greater increases in SAR intensity than surrounding areas, which we used to identify outlines of the emerged areas. The differences in SAR intensity on emerged sandy beaches were not distinguishable compared to the surrounding areas including already existing beaches. Thus, we used orthorectified aerial photographs taken before and after the mainshock to map emerged sandy beaches. The pre-event photographs were taken in 2010 by GSI. The post-event photographs were taken on 2 and 11 January 2024 by GSI (fig. S17). We used the distances between the post- and pre-event coastlines to constrain the advance. In calculating the coastal advance, we ignored small islets and focused on areas with continuous newly emerged land. The pre-event coastline data were those captured in 2017–2023 and were obtained from GSI. The total emerged area was estimated to be 4.5 km^2^. The maximum advance distance was 250 m (Fig. 3). It is difficult to evaluate the estimation error, but the largest-possible error would stem from the tidal variation on a gentle bench or platform slope. A slope of 1% results in up to 50 m of error in the advance estimate considering the tidal range variation of 0.5 m (*63*).

### Survey of the scarps along the Wakayama River Valley

To validate and further investigate surface displacement in the mountain ranges (fig. S18), we conducted field survey along the Wakayama River Valley in the northeastern part of the peninsula. In this area, we used a geological map, landslide inventory, and 1-m mesh digital elevation model for comparison with the field observation (fig. S19).

In the field survey, the amount of displacement was measured using an iPad Pro’s built-in LiDAR sensor; measurements were taken using the Scaniverse application, results were output in LAS format, a raster file of elevation data was created using CloudCompare application, and the displacement on the ground surface was read using the profile tool in QGIS. Most of the scarps showed south-up displacement, but some of them showed north-up displacements. Displacement was particularly concentrated in the Naka area, where linear scarps and multiple scarps appeared in the valley, some of which coincided with pre-existing scarps (figs. S20 to S22). The largest south-up and north-up displacements of 2.2 and 1.3 m, respectively, were identified in the Naka area. East and west of the Naka area, surface deformation was also observed at Munesue and Saneyoshi areas. In both areas, no distinct scarps appeared, but rice paddies were warped (amplitude of less than 1 m).

No large-scale landslides were identified during a survey of the north- and south-facing slopes of the Naka area. A presumed active fault with an east-west strike has been identified on the southern flank of the Wakayama River Valley (*65*), but the SAR pixel offset analysis result does not indicate any clear fault displacement (fig. S18) nor could we observe any distinct surface displacement along the fault trace in the field. Therefore, we infer that this fault was not activated by the 2024 event.

## Supplementary Materials

### The PDF file includes:

Figs. S1 to S23 Tables S1 to S5 Legends for data S1 to S8 References
