# Supplementary material for: Landscape changes caused by the 2024 Noto Peninsula earthquake in Japan
Source: Sci Adv. 2024 Dec 4;10(49):eadp9193. doi: 10.1126/sciadv.adp9193 (PMC11616706; doi:10.1126/sciadv.adp9193)
Supplement: Supplementary file 1 — Figs. S1 to S23 Tables S1 to S5 Legends for data S1 to S8 References [file sciadv.adp9193_sm.pdf]

Science Advances
AAAS

Supplementary Materials for

Landscape changes caused by the 2024 Noto Peninsula earthquake in Japan

Yo Fukushima *et al.*

Corresponding author: Yo Fukushima, yo.fukushima.c3@tohoku.ac.jp

Sci. Adv. **10**, eadp9193 (2024)
DOI: 10.1126/sciadv.adp9193

The PDF file includes:

Figs. S1 to S23
Tables S1 to S5
Legends for data S1 to S8
References

Other Supplementary Material for this manuscript includes the following:

Data S1 to S8

Supplementary Figures and Tables

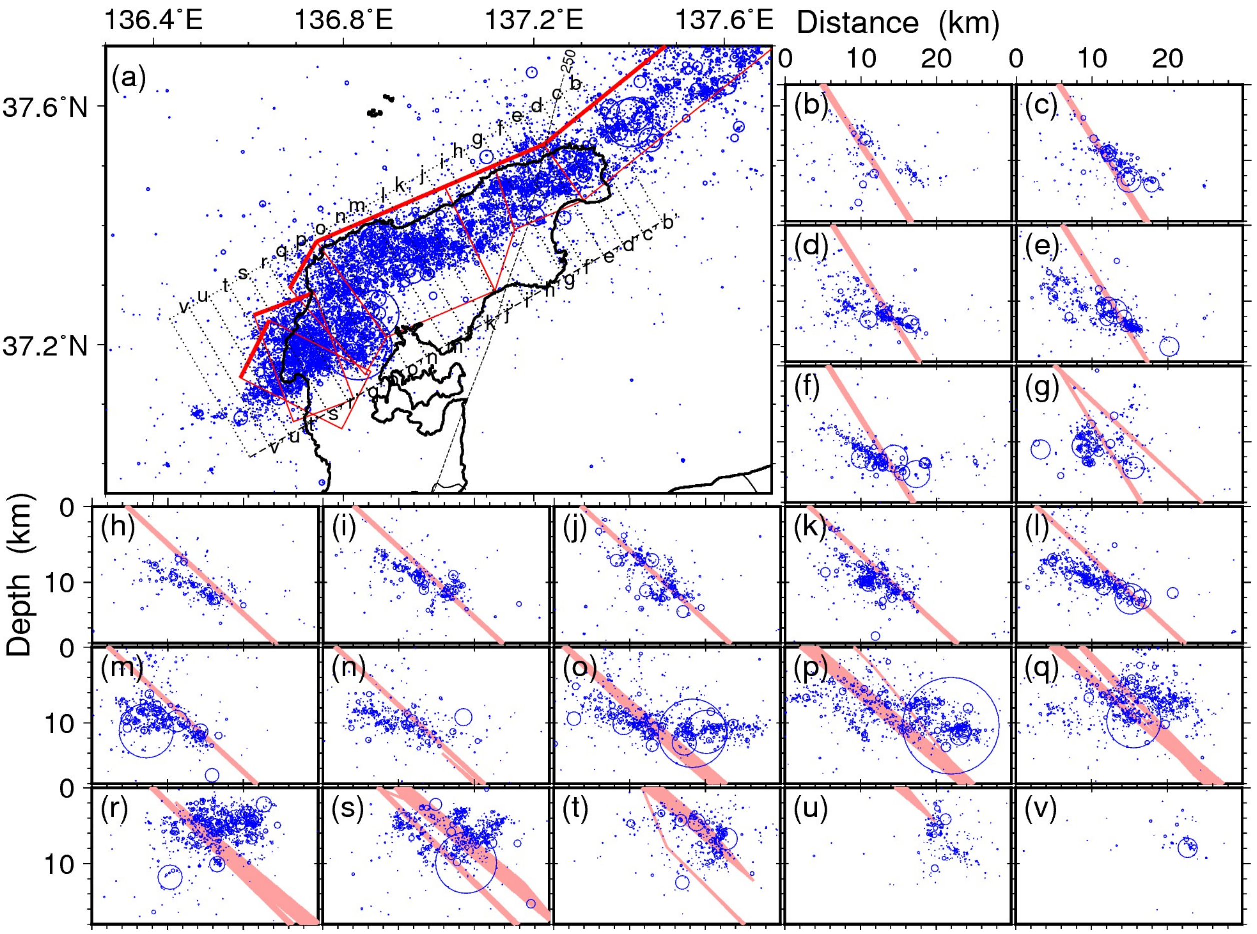

Fig. S1. Relocated aftershock distribution.
(a) Map view. (b)-(v) Cross-sectional view. Circles represent the aftershock distribution, and their sizes correspond to typical fault sizes based on a stress drop of 3 MPa. The red lines denote the model faults adopted in this study (Section 3 of Materials and Methods).

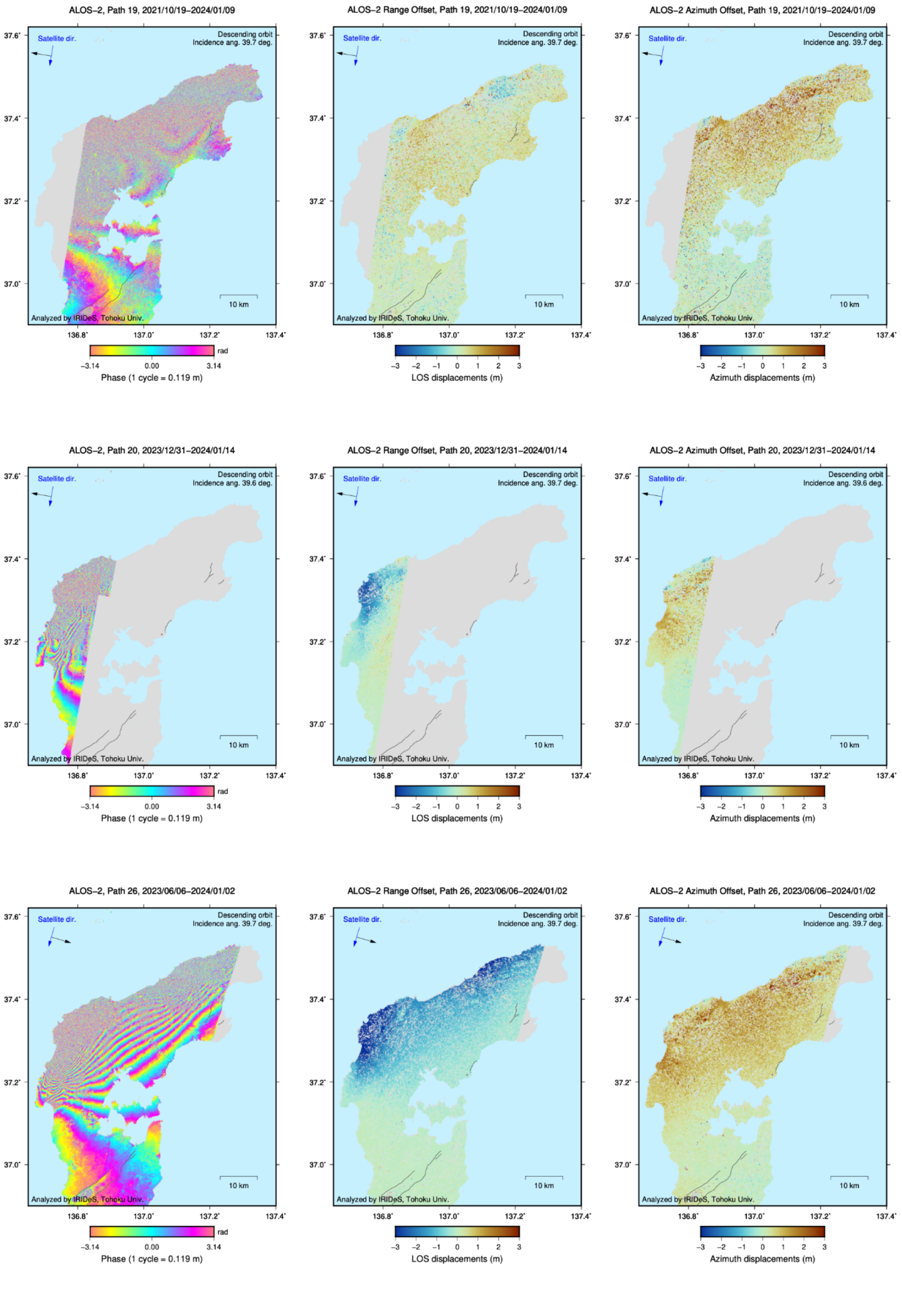

Figure S2. Deformation observed from SAR data analysis.
SAR interferogram (left), range offset displacements (middle), and azimuth offset displacements (right) obtained and used in this study.

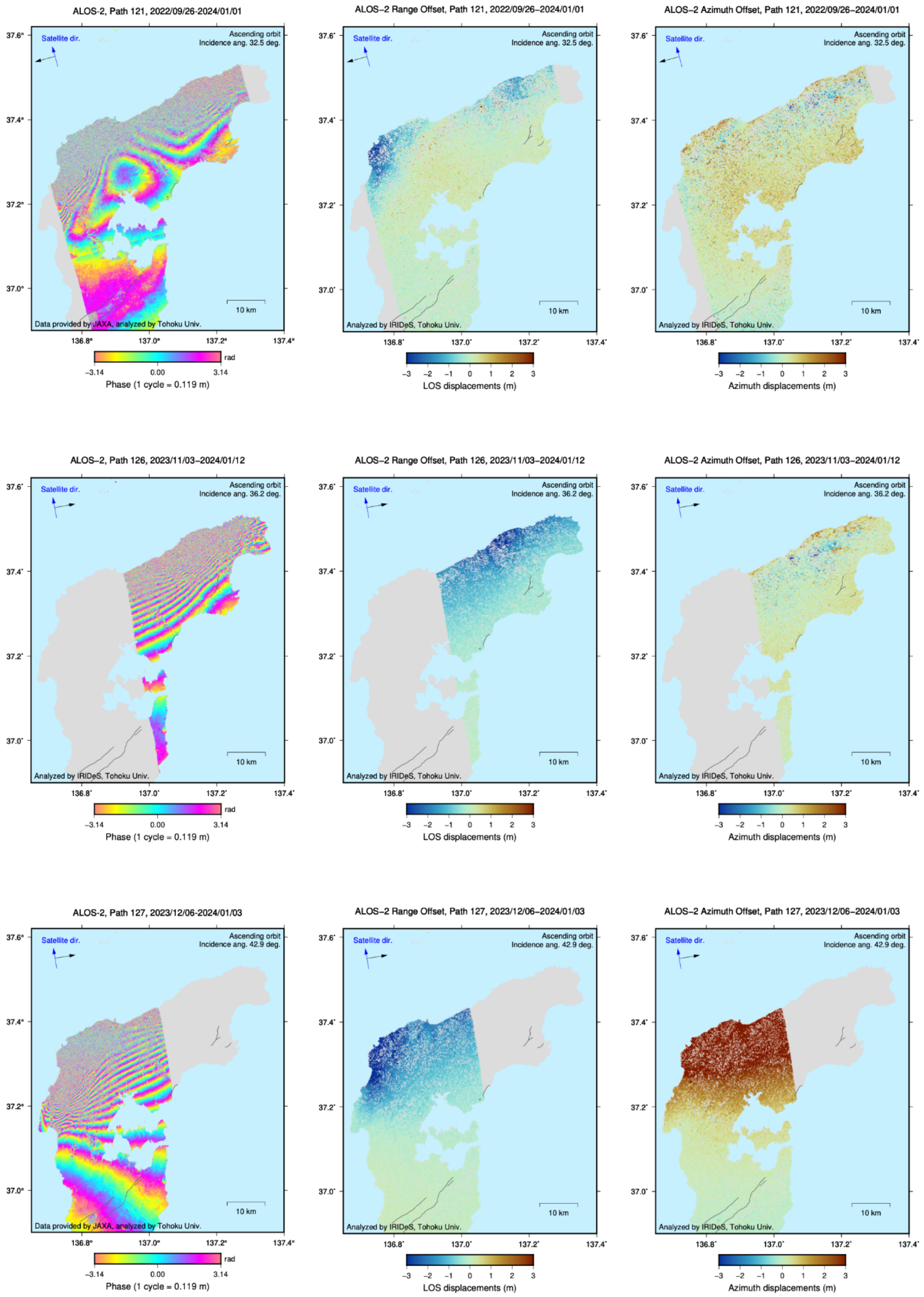

Figure S2 continued.

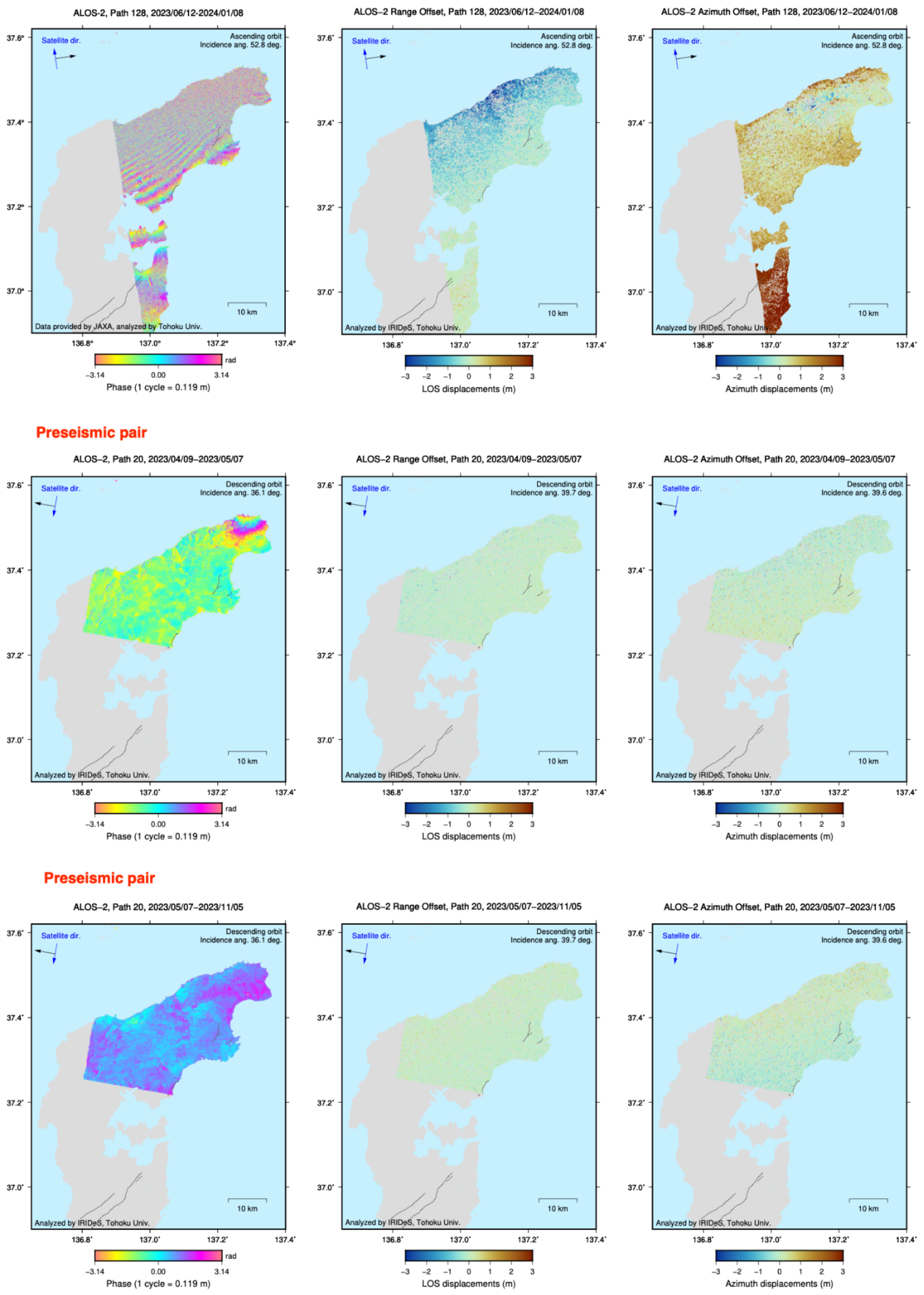

Figure S2 continued.

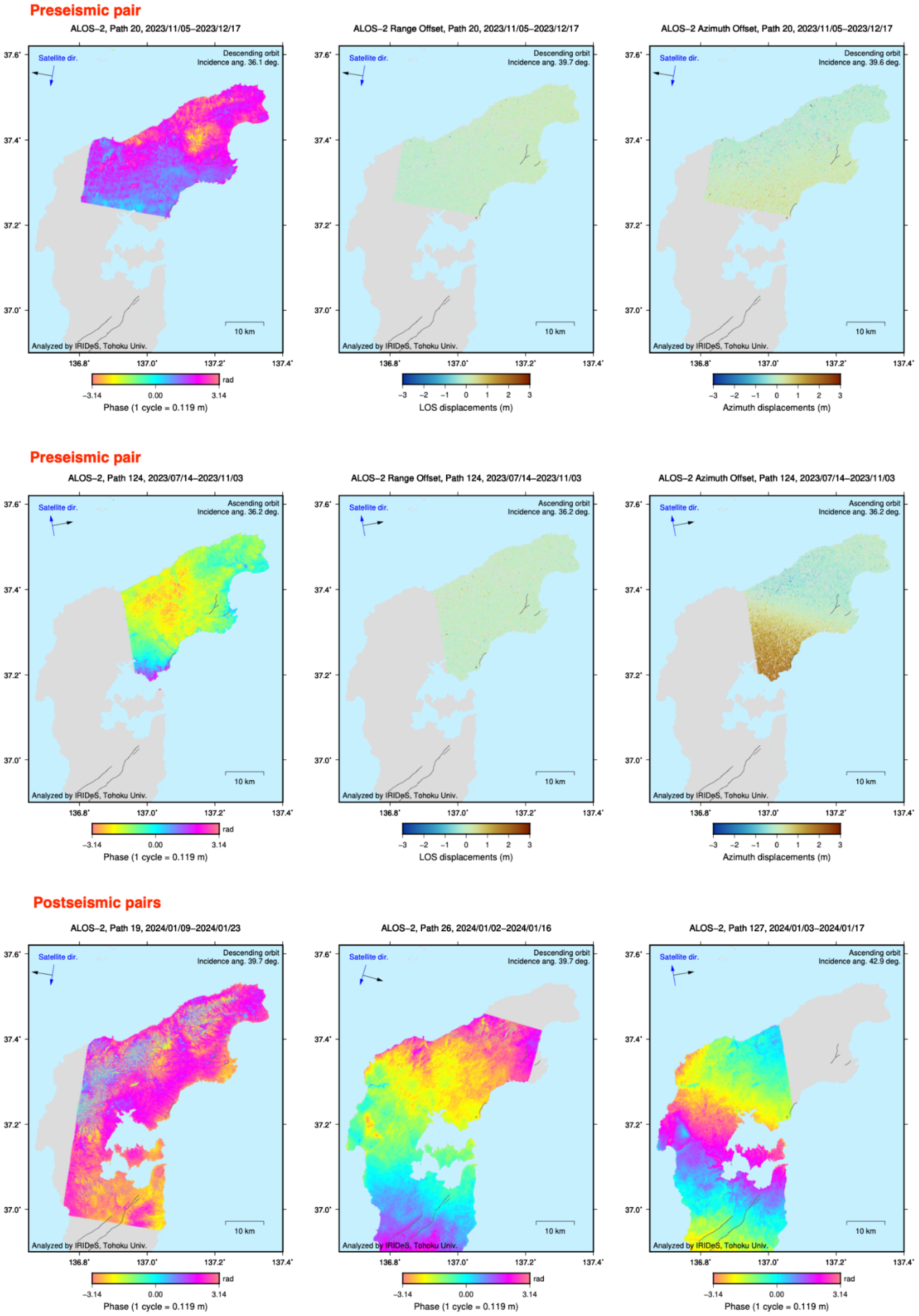

Figure S2 continued. The bottom row shows the postseismic SAR interferograms from Paths 19, 26, and 127.

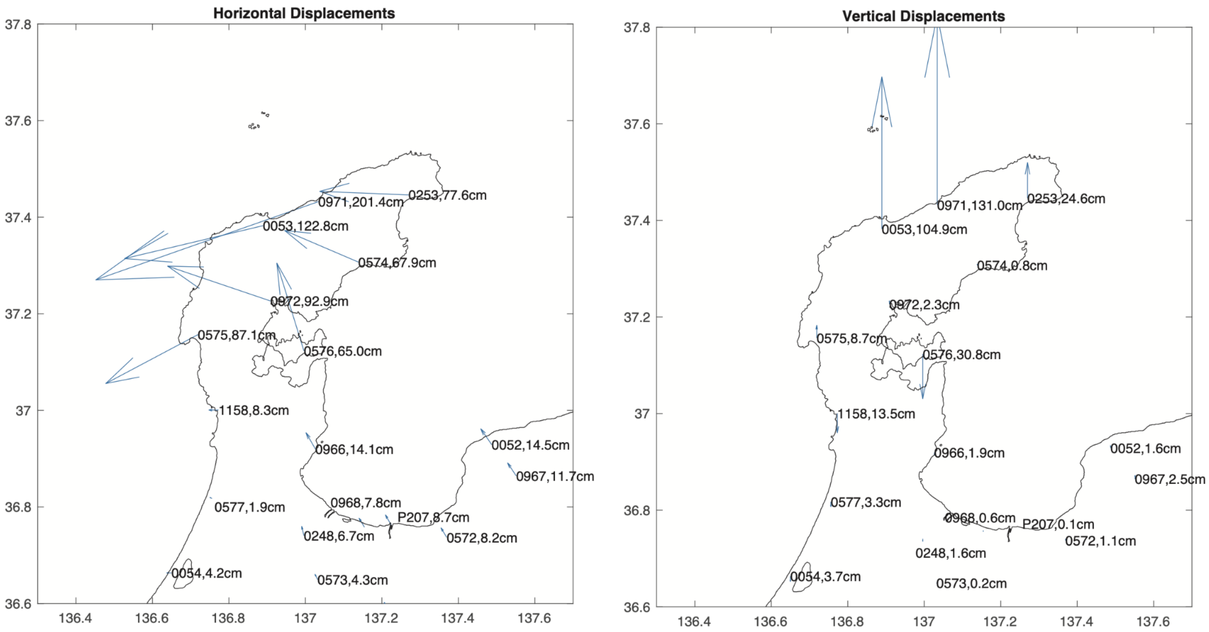

Figure S3. GNSS coseismic displacements.
The coseismic displacements were extracted from the F5 solution of the GEONET GNSS observation network.

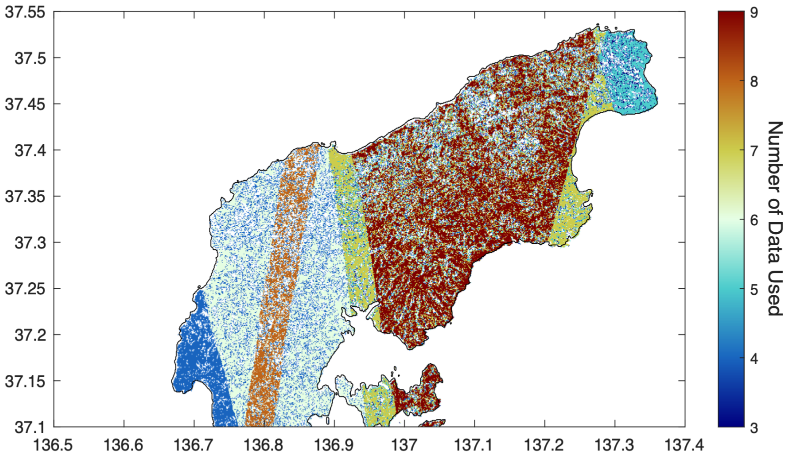

Figure S4. Number of data (displacements from different directions) used for the 3D decomposition of the displacements.

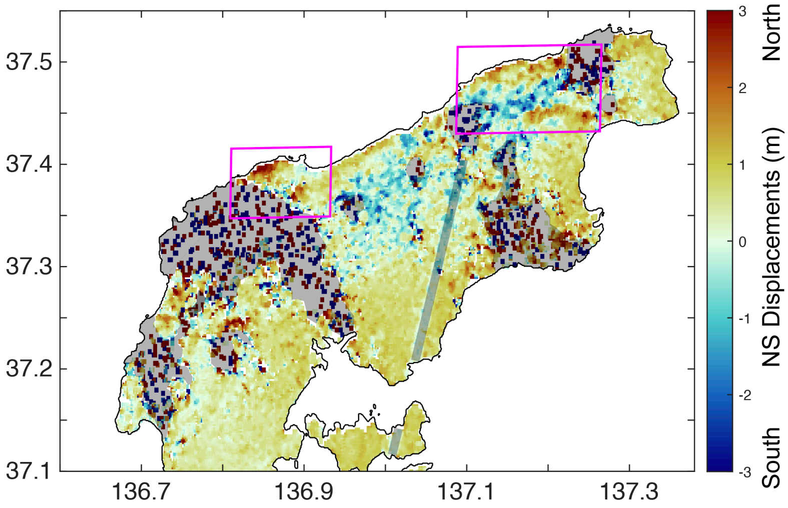

Figure S5. North-south displacements obtained from the pixel offset analysis using Sentinel-2 satellite data and COSI-Corr software.
Magenta rectangles show areas of hillslope slumps. Areas of low confidence are masked with gray shades.

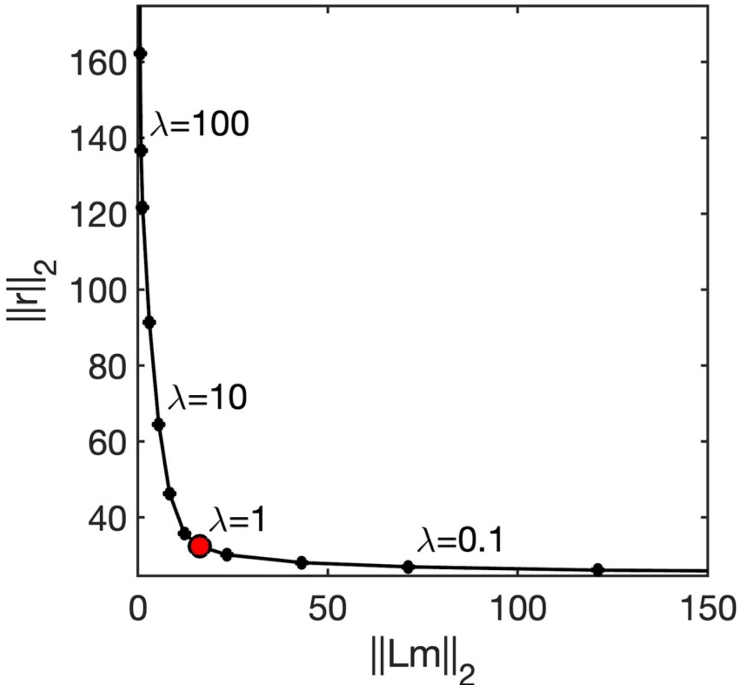

Figure S6. Tradeoff between the model roughness and misfit.
The x and y axes correspond to the roughness and misfit, respectively. The results were obtained with different weights of the regularization. The red dot marks the model adopted in this study.

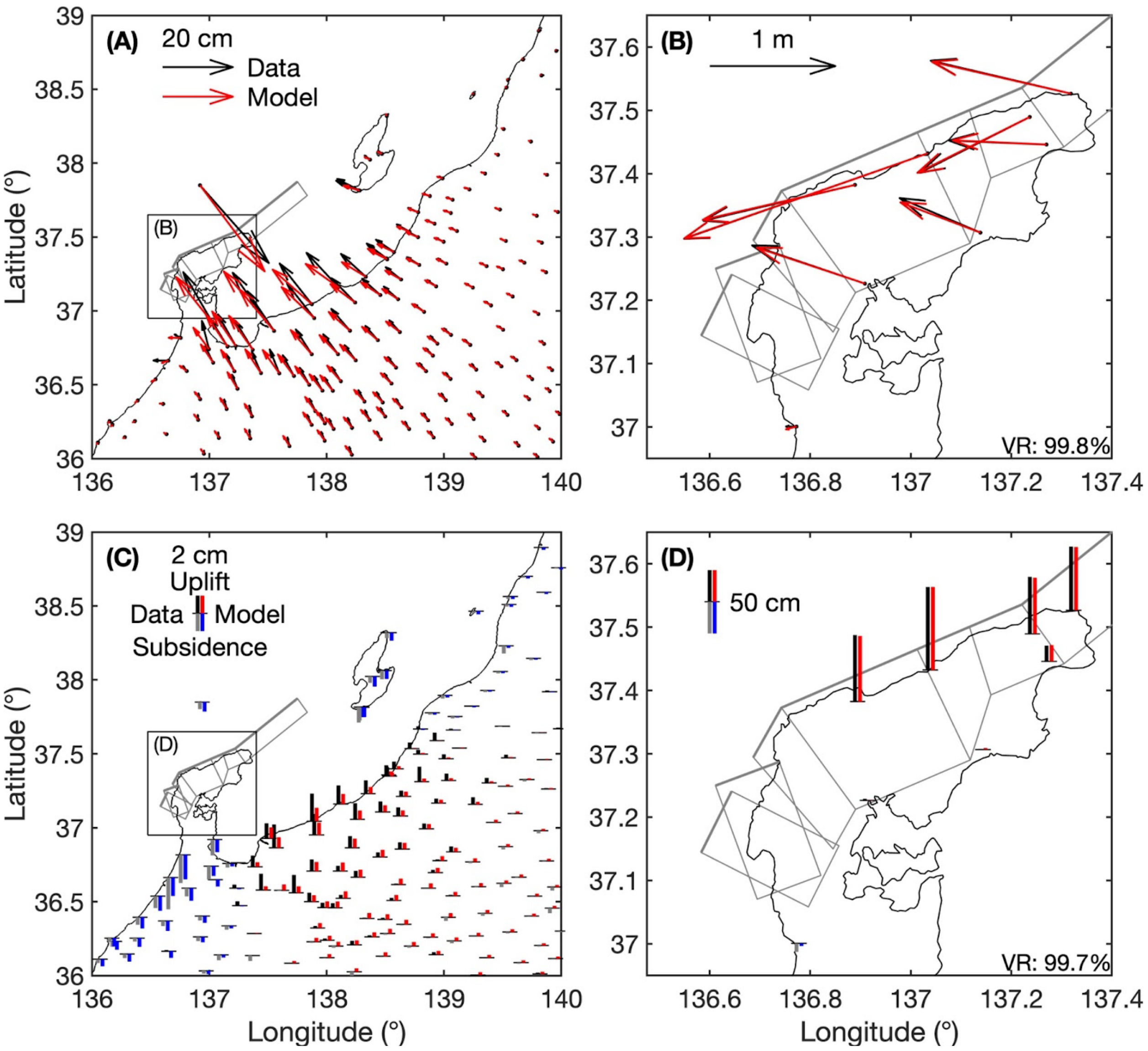

Figure S7. Observed and modeled GNSS static coseismic displacements.
(A, B) Horizontal displacements. (C, D) Vertical displacements. (B) and (D) are a zoomed-in view of the large displacements observed on the Noto Peninsula with vectors in smaller scales. Gray polygons outline the fault geometry.

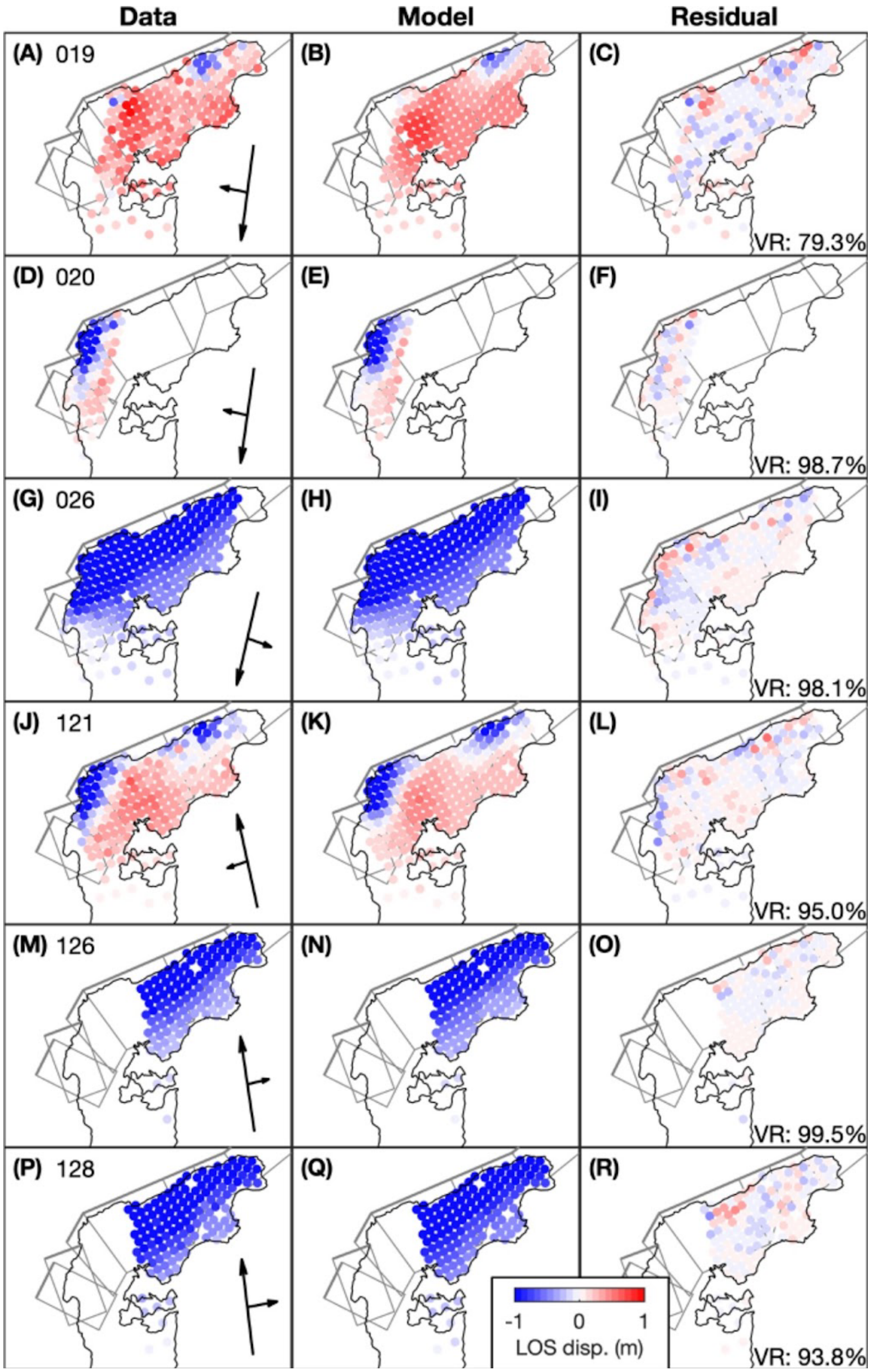

Figure S8. Observed, modeled, and residual range offsets.
The results for Paths (A-C) 19, (D-F) 20, (G-I) 26, (J-L) 121, (M-O) 126, and (P-R) 128 are shown. In the observed offsets, bilinear ramps are removed. Blue and red indicate motion toward and away from the satellite, respectively. Gray polygons outline the geometry of the fault.

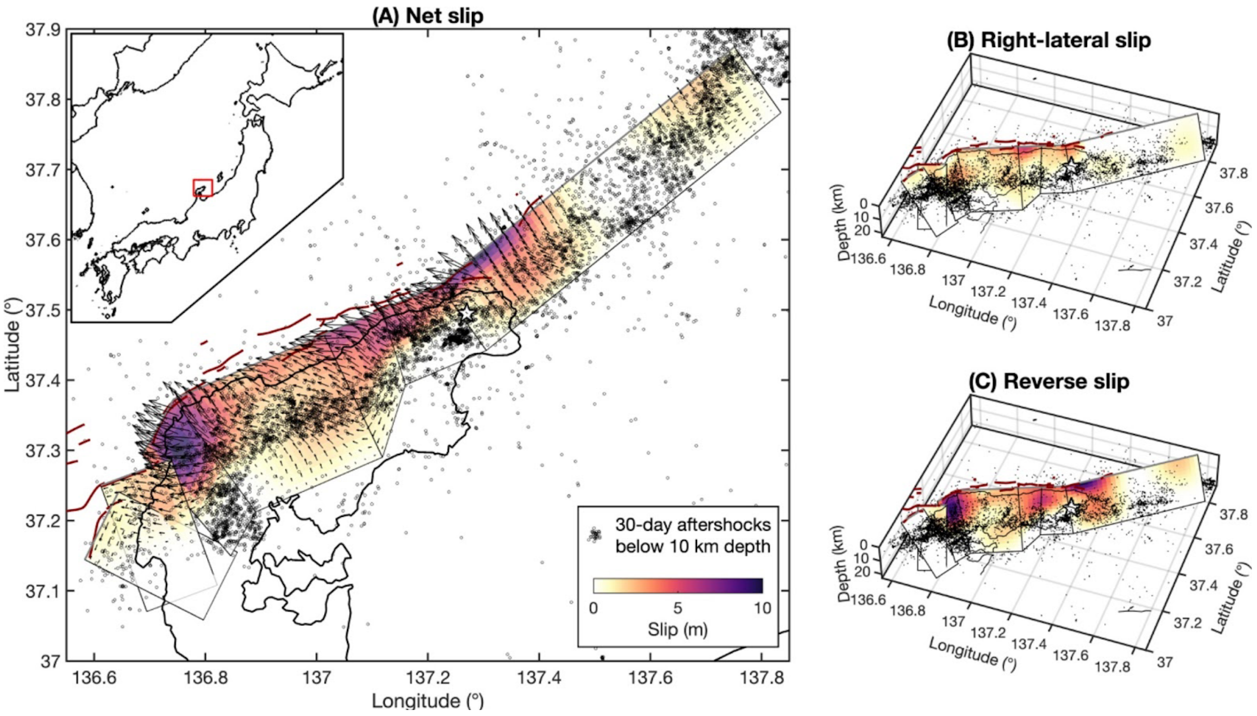

Figure S9. Coseismic slip model.
(A) Net slip, (B) right-lateral component of slip, and (C) reverse component of slip. White star indicates the hypocenter of the 2024 earthquake. Black dots are the 30-day relocated aftershocks, while in (A) we highlighted the aftershocks below 10 km depth. Black vectors show the slip vectors weighted by the amount of slip.

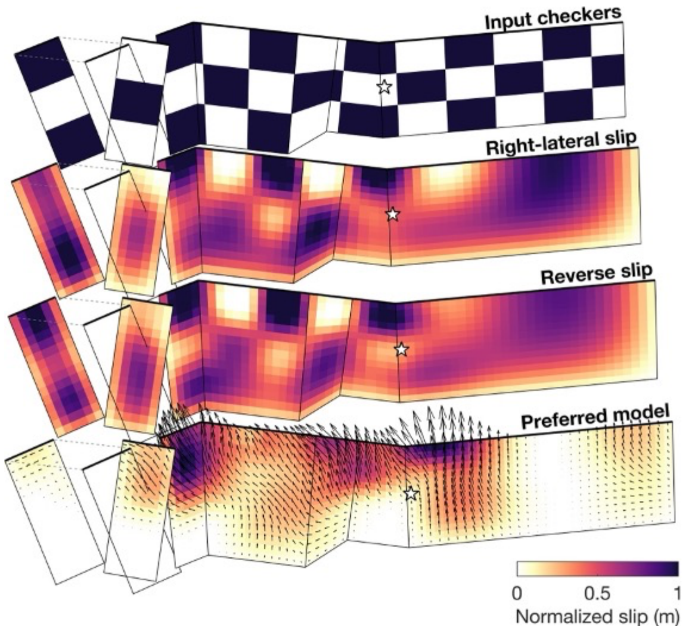

Figure S10. Checkerboard test using input patterns consisting of 6-by-5 fault elements. From top to bottom are the input checkers, recovered right-lateral slip, recovered reverse slip, and our preferred model. The southwestern-most fault segment is shifted for a better visualization.

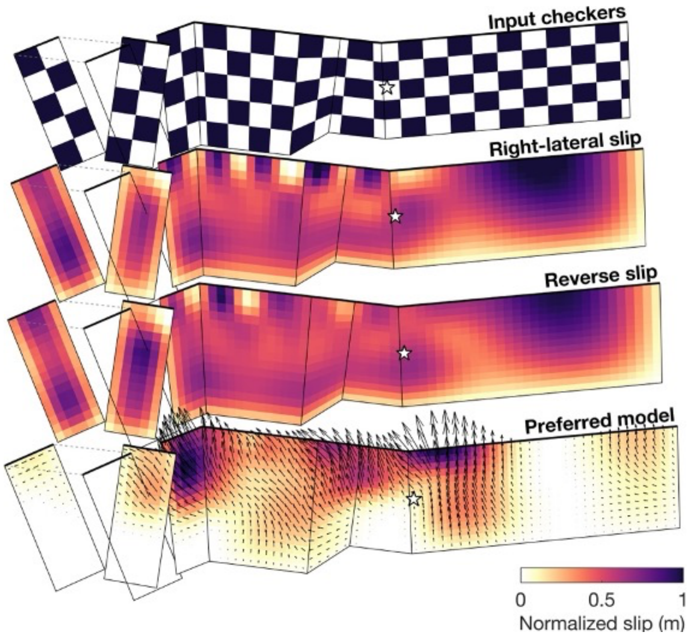

Figure S11. Checkerboard test using input patterns consisting of 3-by-3 fault elements. From top to bottom are the input checkers, recovered right-lateral slip, recovered reverse slip, and our preferred model. The southwestern-most fault segment is shifted for a better visualization.

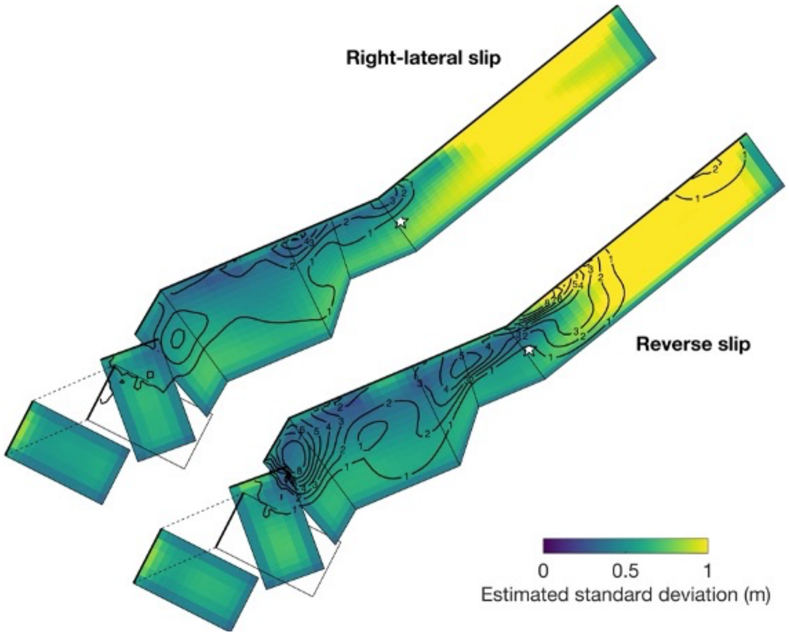

Figure S12. Estimated standard deviations of the inverted coseismic slip.
The results are shown independently on strike-slip and dip-slip components. Black contours indicate the amount of slip in meters at 1 m intervals. The maximum standard deviations of the strike-slip and dip-slip components are 1.6 and 1.7 m, respectively, in the northeastern segment with saturated colors. The southwestern-most fault segment is shifted for a better visualization.

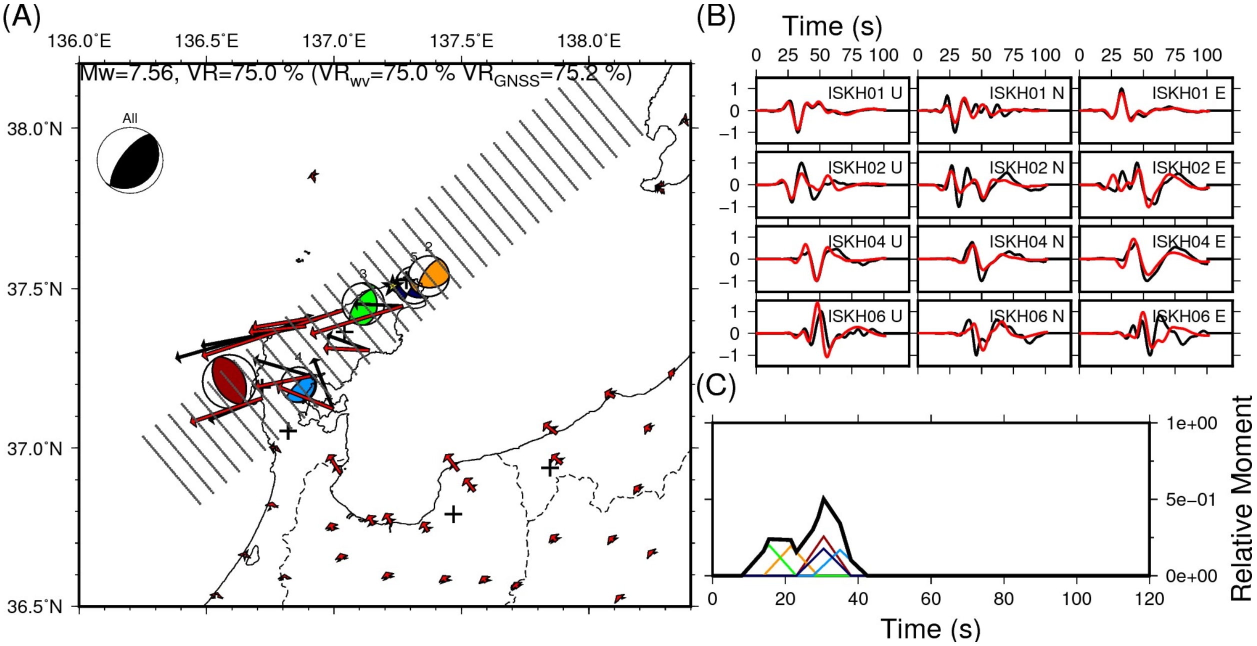

Figure S13. Sub-events estimated using the method of (*60*).
(A) Spatial distribution of the moment tensor of the sub-events 1 to 8. Black and red arrows represent observed and calculated horizontal surface displacements, respectively, and crosses represent strong-motion observation points. The mechanism solution in the upper right represents the sum of all sub-events. (B) Comparison of observed (black) and synthetic (red) strong-motion waveforms. (C) Relative moment-rate function. Colors correspond to the respective sub-events, and black is the sum.

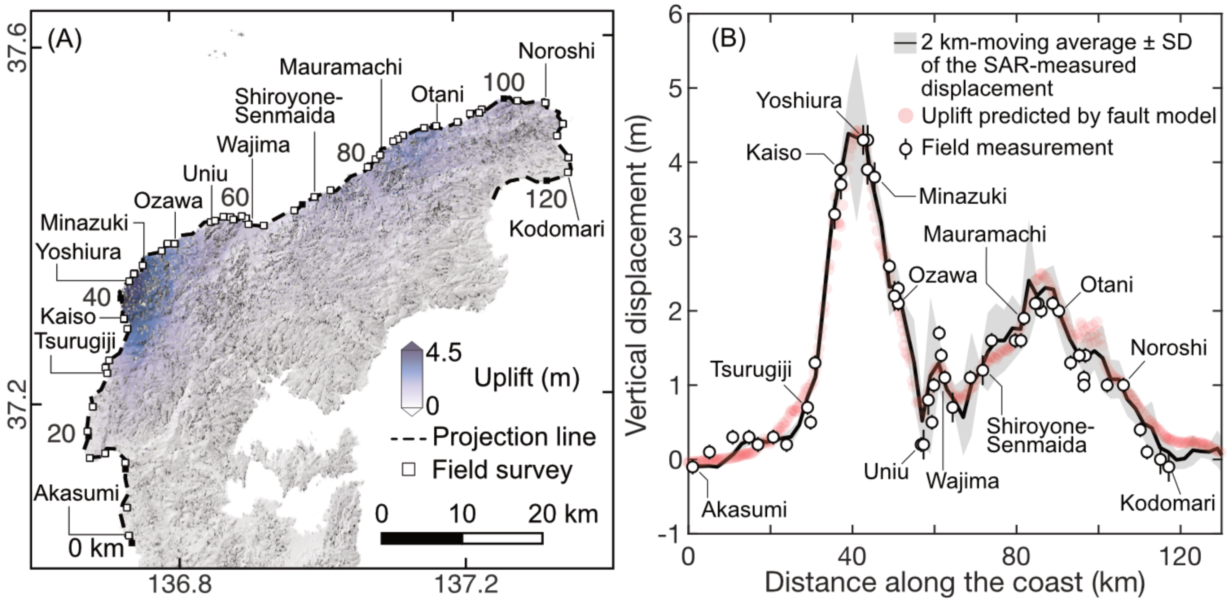

Figure S14. Comparison of vertical displacements of SAR and field measurements.
(A) Vertical displacement derived from the SAR pixel offset analysis and sites of field survey. Vertical displacement in (B) is projected onto the broken line. The numbers along the coast indicate the distance in (B). (B) Vertical displacement along the coast. Data points within 300 meters from the projection line in (A) are used.

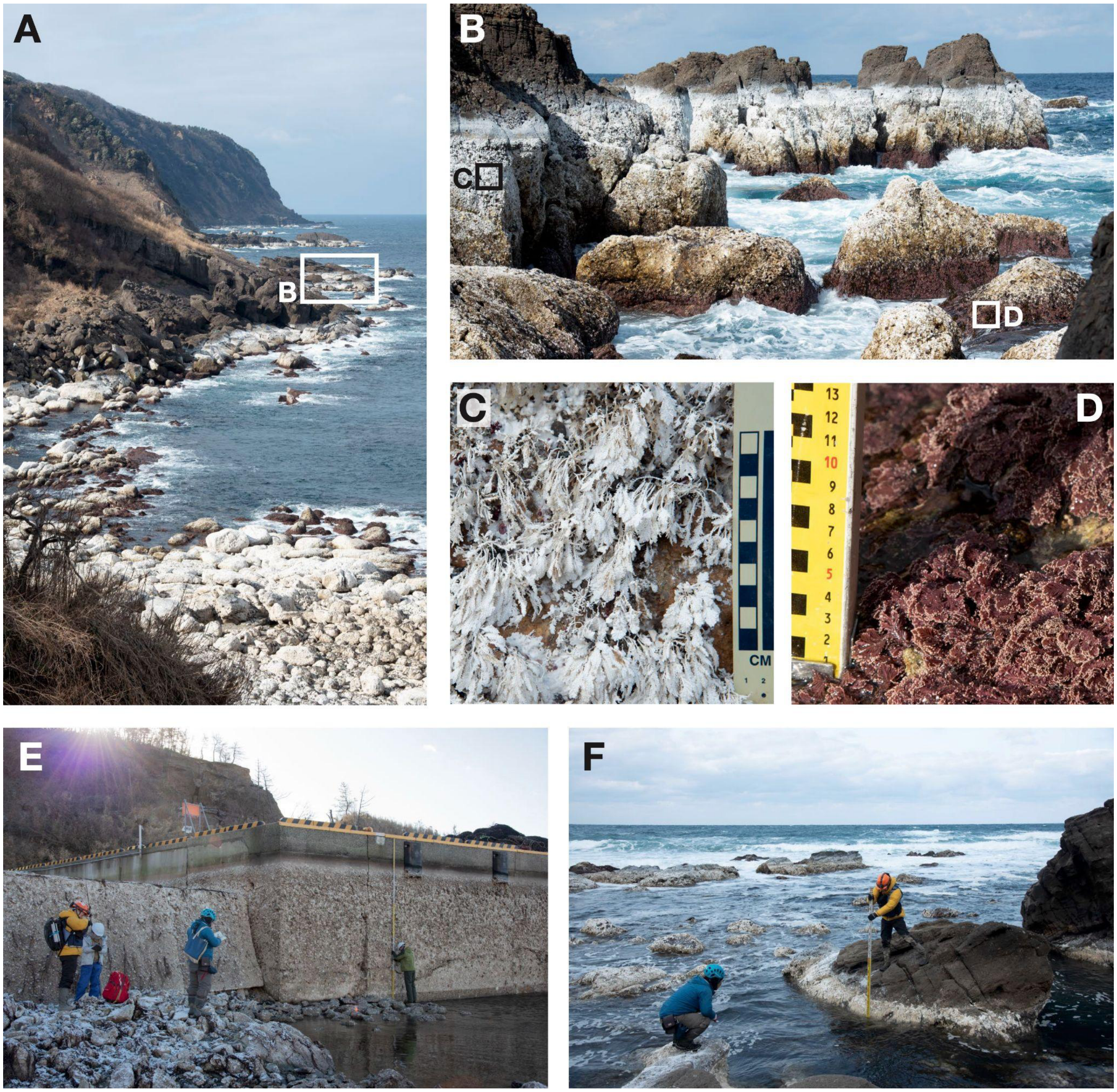

Figure S15. Evidence of coastal uplift along the North Noto coastline and explanation of the survey method.
(A) Noto coastline between Kamiozawa and Ozawa in Wajima. (B) White band of dead pirihiba calcareous red algae uplifted out of the water by the earthquake. Inset indications show approximate areas of the pictures C and D. (C, D) Close-ups of dead and alive pirihaba red algae, respectively. (E) Uplift survey in Kaiso Port (ID#6) using oyster. (F) Uplift survey at Mitanimachi (ID#46) using pirihiba, 1 km west of Hikariuria in Wajima. Photo Credit: (A-F) Daisuke Ishimura, Tokyo Metropolitan University.

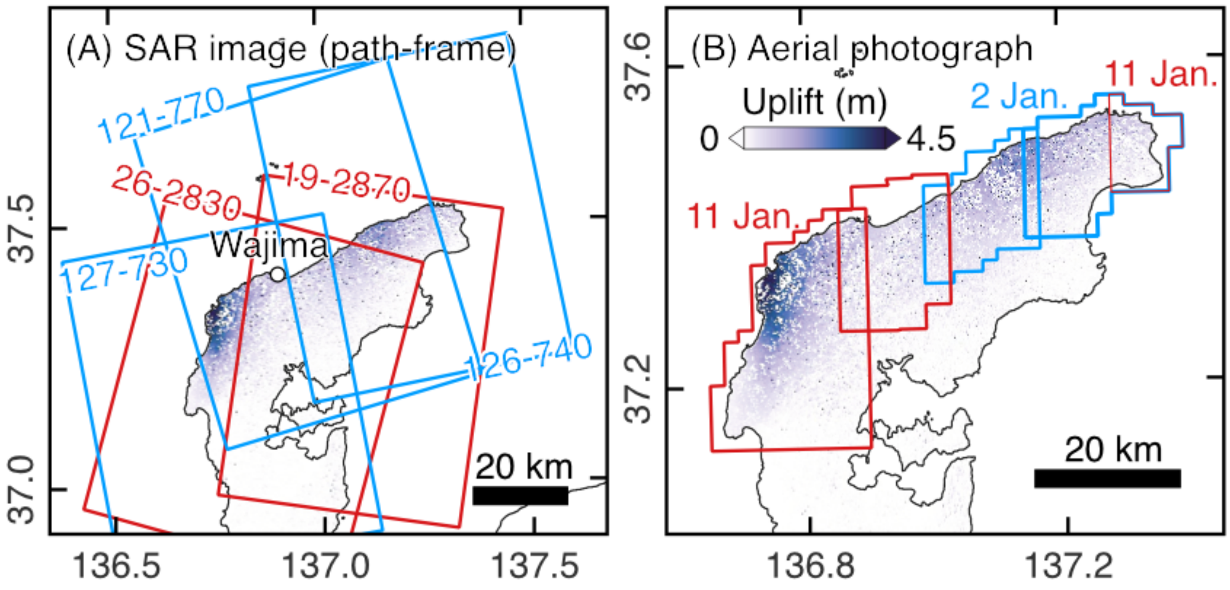

Figure S16. SAR scene and aerial photograph coverage.
(A) SAR scene coverage and (B) aerial photograph coverage used to map the emerged areas along the coast.

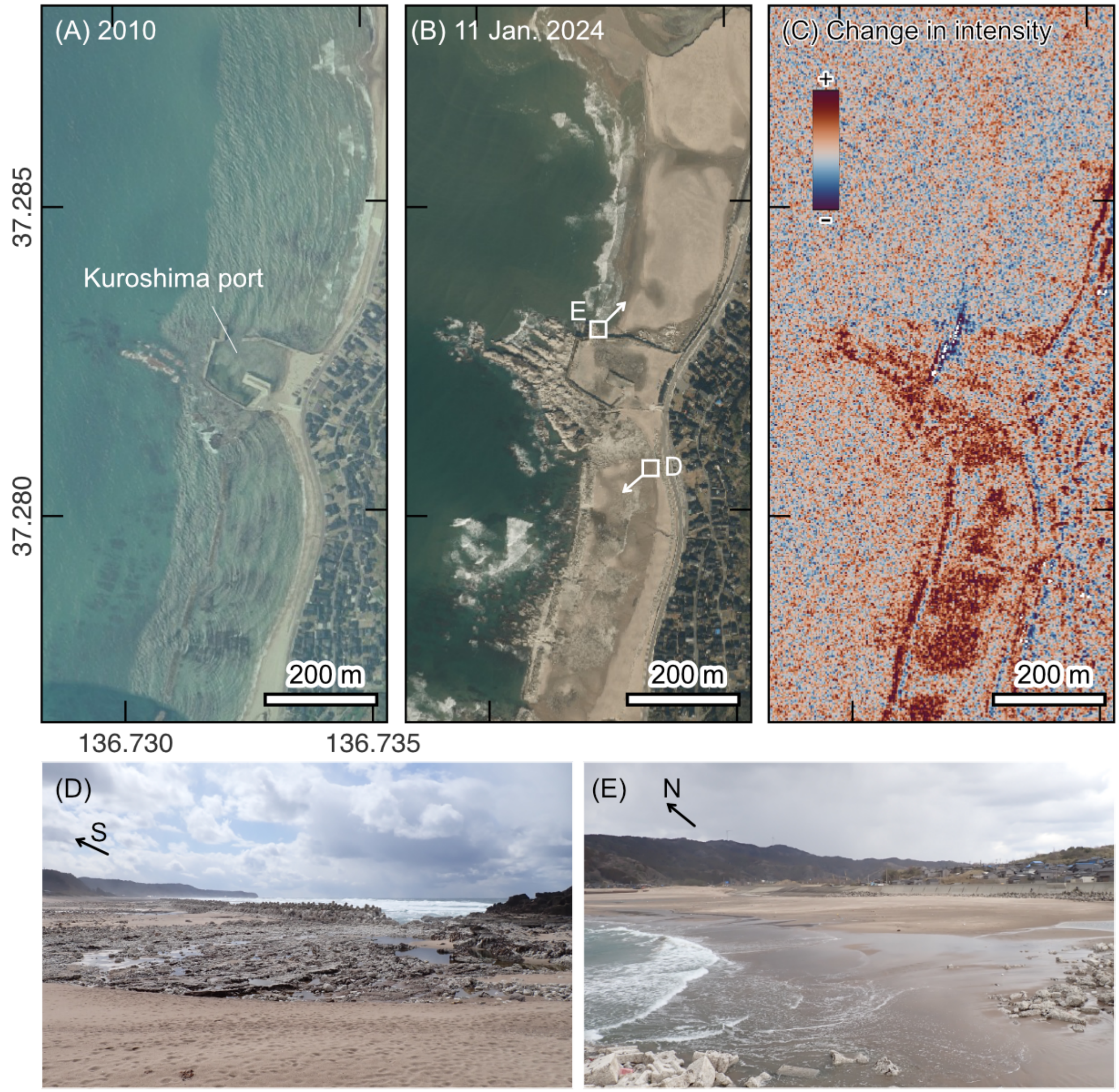

Figure S17. Change of the beaches at Kuroshima Port.
(A, B) Rocky and sandy beaches at Kuroshima Port before and after the 2024 earthquake, respectively. (C) The changes in intensity of SAR images taken before and after the 2024 earthquake (Path 26). (D) Rocky beach south of Kuroshima Port. (E) Sandy beach north of Kuroshima Port. Photo Credit: (A, B) GSI, (D, E) Daisuke Ishimura, Tokyo Metropolitan University.

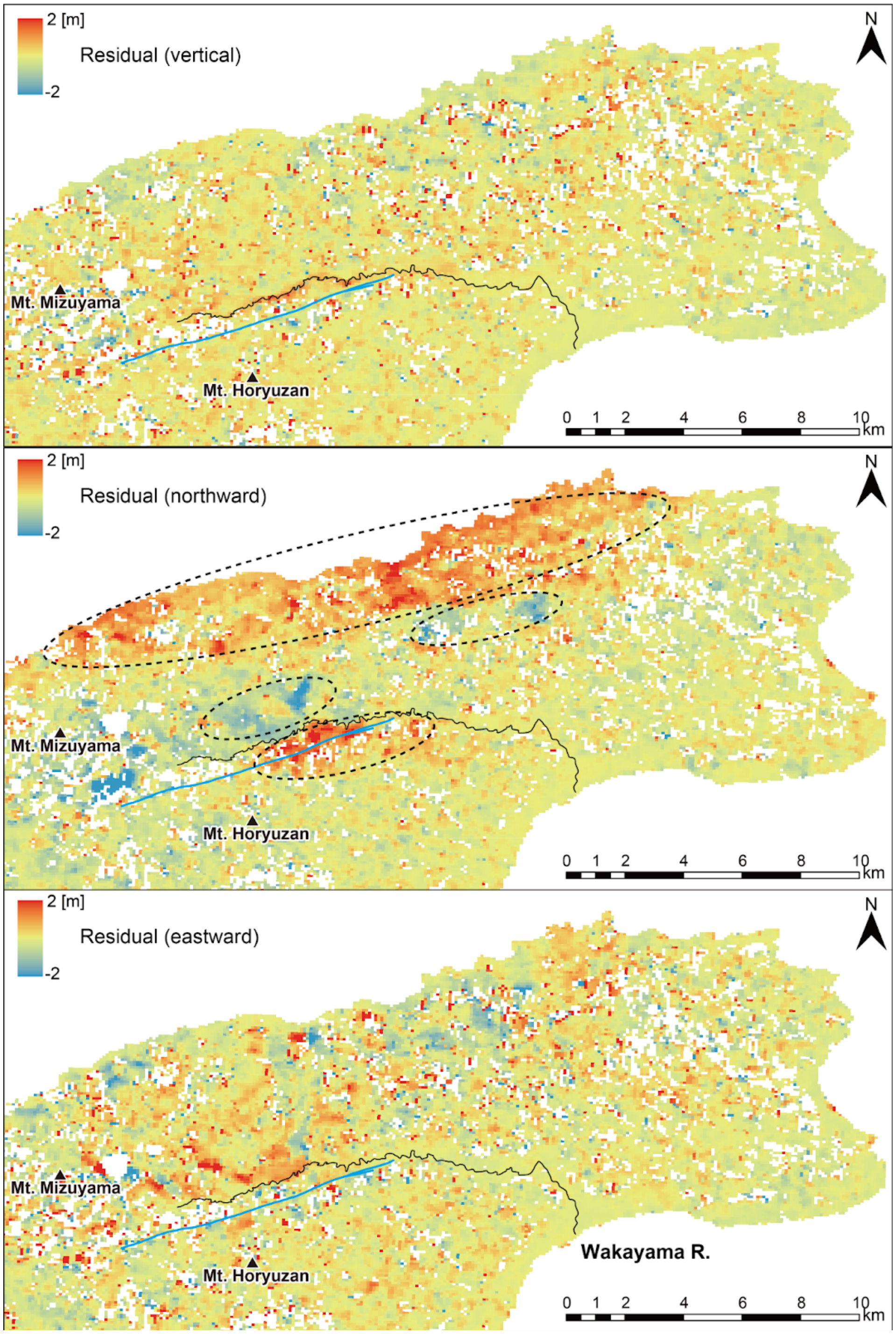

Figure S18. Residual displacement field in the northeastern part of the Noto Peninsula. Blue lines are the presumed active faults (*66*). Black line is the Wakayama River course. Ellipsoids in broken lines in the middle figure indicate hillslope slump signals.

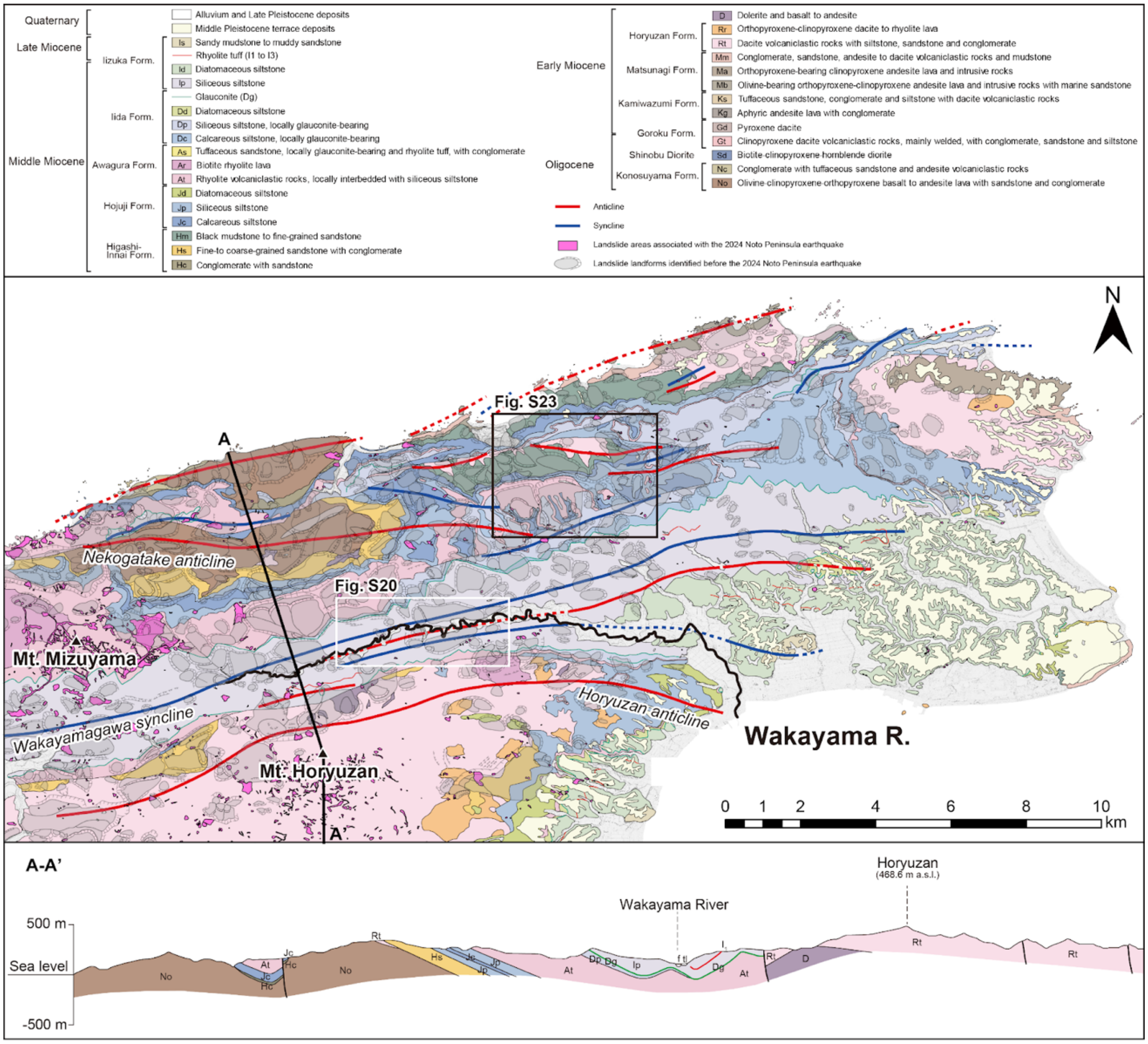

Figure S19. Geology and landslide distribution.
Geology is from vector data (v01) of (*66*). Gray-colored landslides are from (*67*). Pink-colored ones are the landslides triggered by the 2024 Noto Earthquake (*14*). Topological basemap is slope and hillshade maps from 1-m mesh DEM from the Ishikawa Prefecture (*68*).

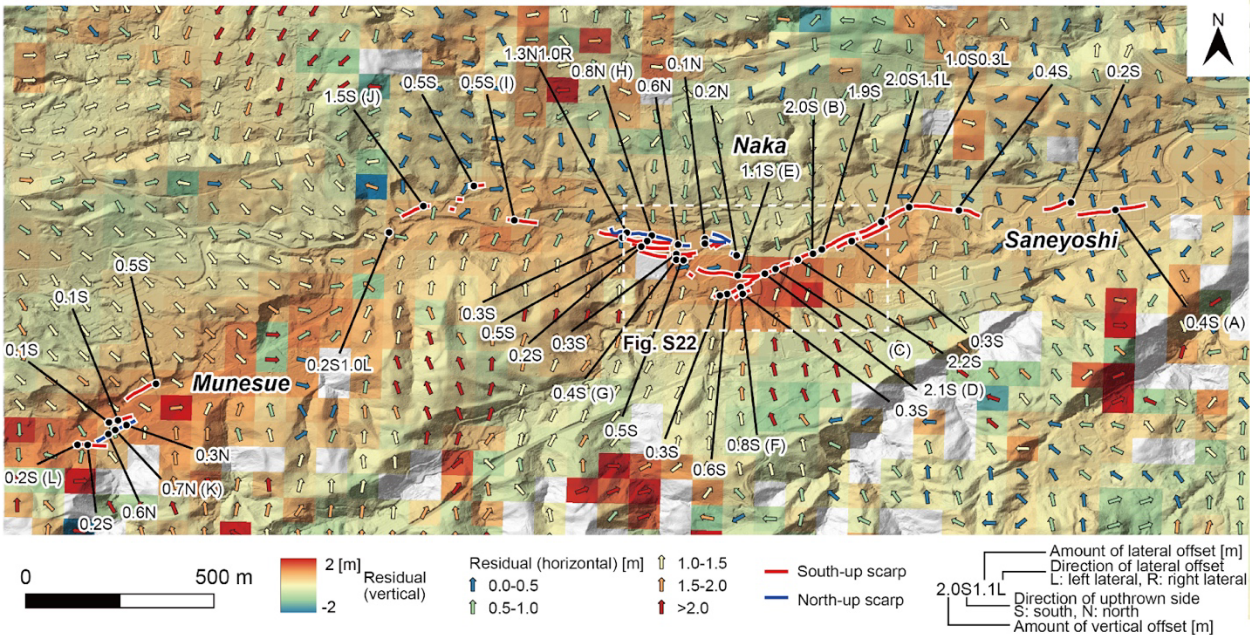

Figure S20. Scarp distributions and displacement measurements.
Residual displacement field data used in this map is the same as Fig. S18. Hill shade map is from 1-m DEM from Ishikawa Prefecture (*68*).

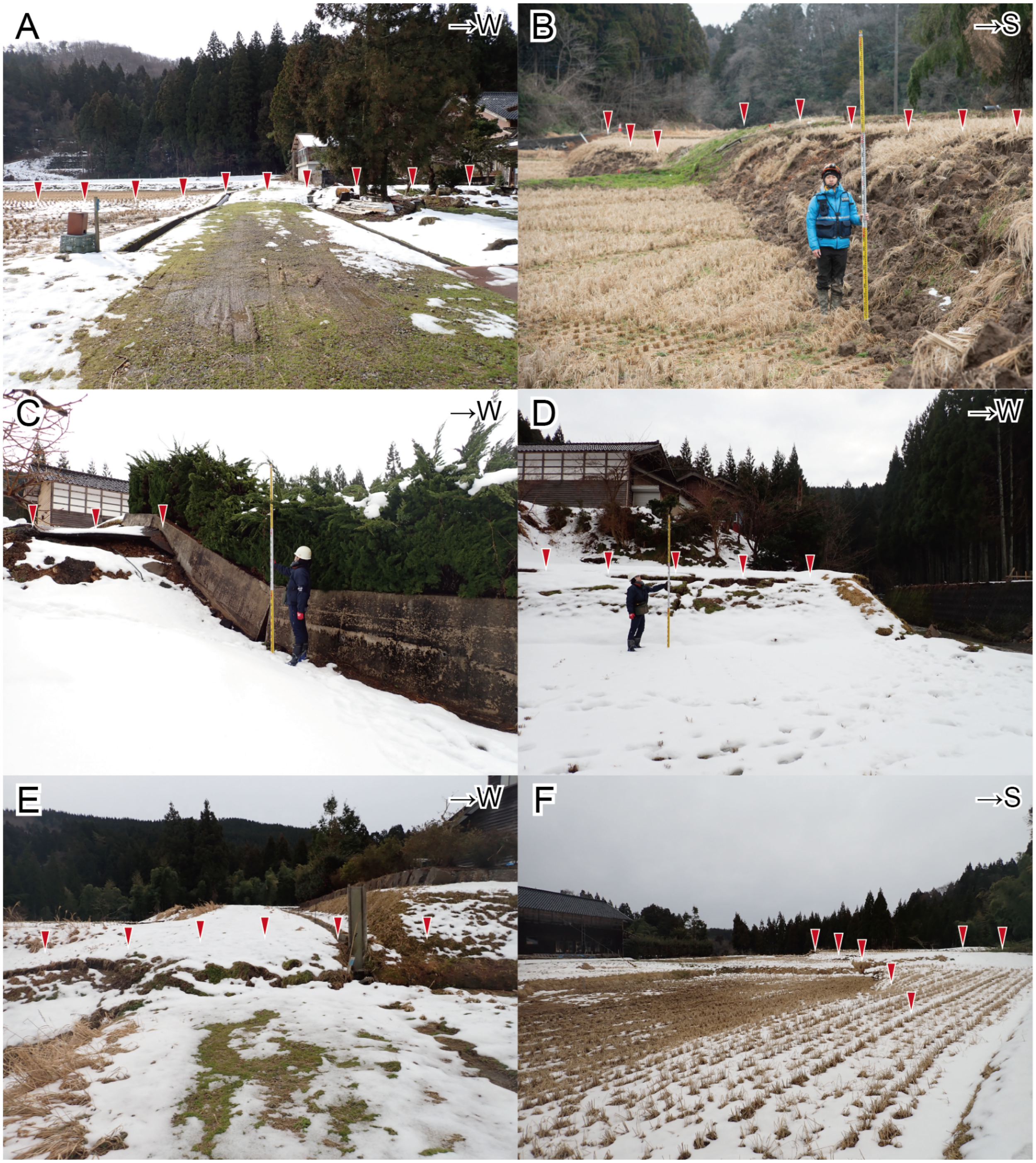

Figure S21. Surface displacements along the Wakayama River.
Locations are shown in Figure S20. Red arrows denote the scarp traces. Photo Credit: (A, C–L) Daisuke Ishimura, Tokyo Metropolitan University. (B) Luca C. Malatesta, GFZ German Research Center for Geosciences.

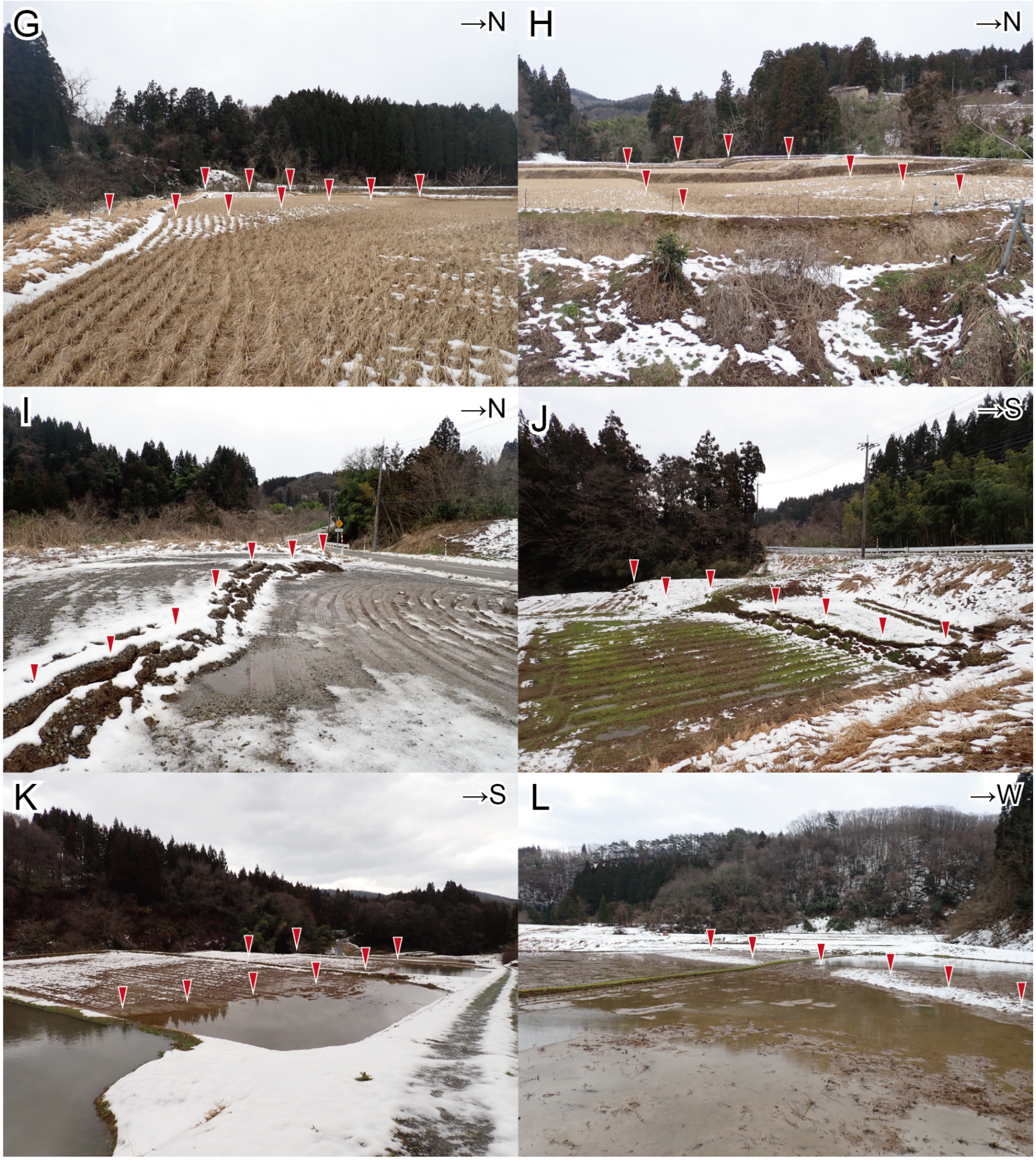

Figure S21 continued.

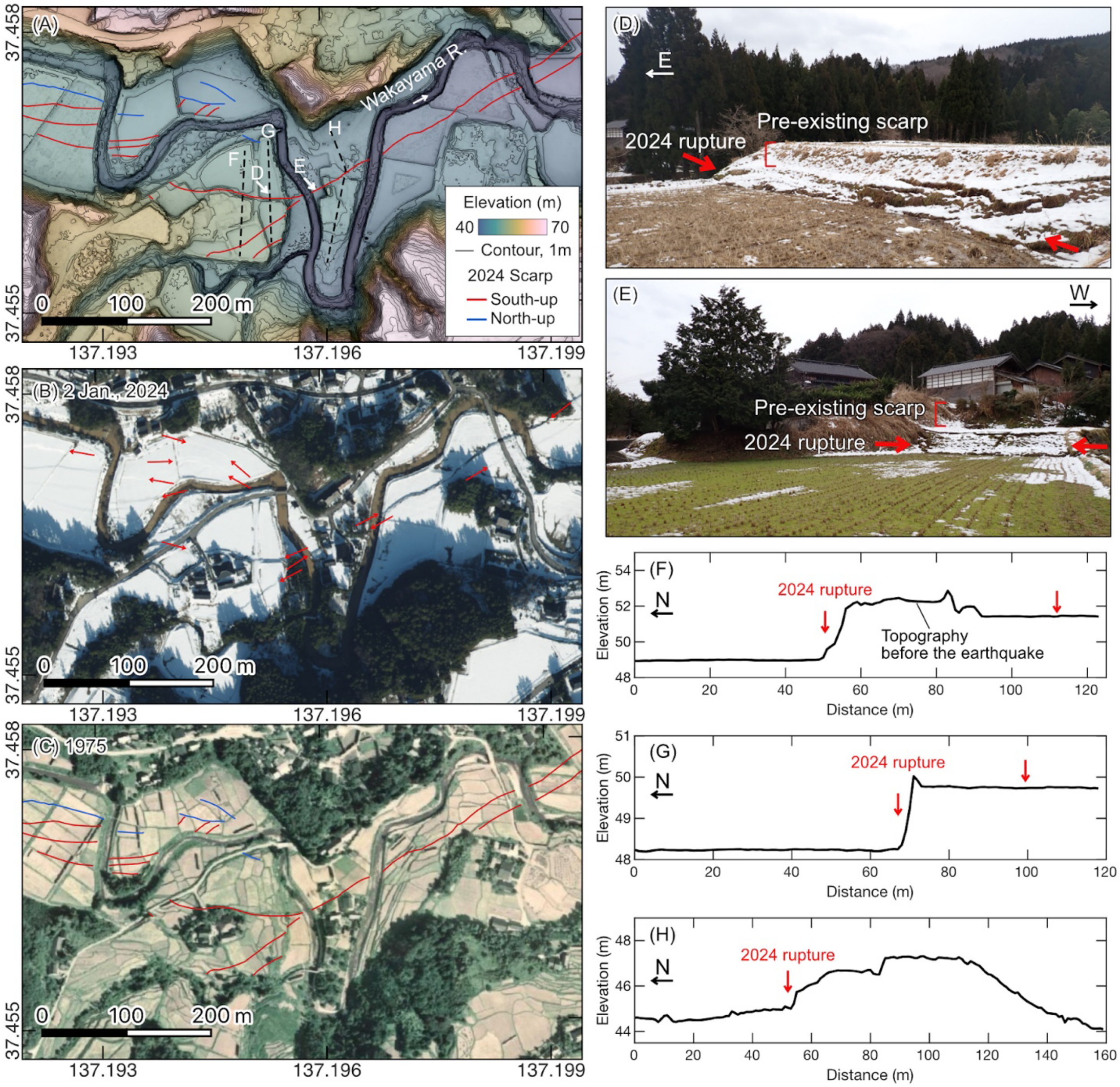

Figure S22. Topography and surface rupture along the Wakayama River.
(A) Topography before the earthquake and the surface rupture of the earthquake. (B) Aerial photograph taken by GSI on 2 January 2024. Red arrows indicate the surface rupture. (C) Aerial photograph taken by GSI in 1975. Red and blue lines are the same as those in (A). (D-E) The surface ruptures occurred at the pre-existing scarp. (F-H) Topographic profiles along the lines F-H shown in (A). Elevation is based on 1-m DEM from Ishikawa Prefecture (*68*). Photo Credit: (B, C) GSI, (D, E) Daisuke Ishimura, Tokyo Metropolitan University.

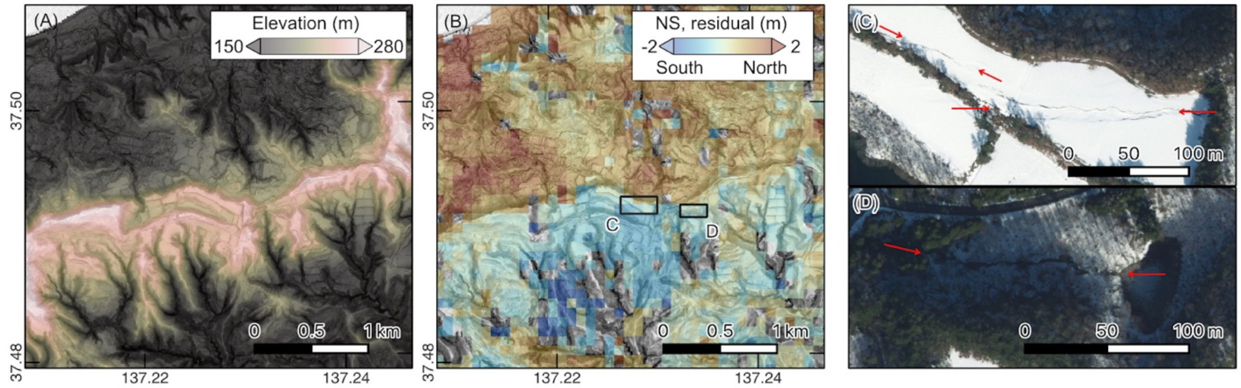

Figure S23. Topography and deformation along a ridge north of the Wakayama River. (A) Topography around a double ridgeline. Elevation is based on 1-m DEM from Ishikawa Prefecture (*68*). (B) North-south residual displacement. (C, D) Aerial photos taken by GSI on 2 January 2024 (*14*). Photo Credit: GSI.

Table S1. SAR images used in this study

ALOS-2 orbit path number	Incidence Angle (deg)	Looking direction	Acquisition dates before and after the earthquake (YYYY/MM/DD)	RMS of the difference with the GNSS displacement (cm)		Used in the 3D displacement decomposition	
				Range	Azimuth	Range	Azimuth
19	39.7	ESE	2021/10/19−2024/01/09	16.8	6.6	Yes	Yes
20	39.6	ESE	2023/12/31−2024/01/14			Yes (bias adjusted with other datasets)	Yes (bias adjusted with other datasets)
26	39.7	WNW	2023/06/06−2024/01/02	8.8	22.6	Yes	Yes (bias manually adjusted)
121	32.5	ENE	2022/09/26-2024/01/01	5.5	10.8	Yes	Yes
126	36.2	WSW	2023/11/03−2024/01/12	7.8	8.0	Yes	Yes
127	42.9	WSW	2023/12/06-2024/01/03	18.0	205.7		
128	52.8	WSW	2023/06/12-2024/01/08	0.4	28.7	Yes	

Table S2. RMS error and variance reduction (VR) for each geodetic dataset

	GNSS			ALOS-2 range offsets for different Path numbers					
	E	N	U	19	20	26	121	126	128
RMS (cm)	1.07	0.59	0.95	17.81	14.57	12.54	13.31	8.33	13.70
VR (%)	99.8	99.3	99.7	79.3	98.7	98.1	95.0	99.5	93.8

Table S3. Overall data variance reduction (VR) obtained for different models

Model	VR (%)
Uniform dip angle 40°	97.07
Uniform dip angle 45°	97.12
Uniform dip angle 50°	96.90
Listric: 60° (0-10 km) and 30° (10-20 km)	96.53
Preferred model (dip varying along strike)	97.16

Table S4. Comparison of focal mechanisms

	Strike (°)	Dip (°)	Rake (°)	Mw
NIED	210/48	45/46	78/102	7.5
USGS	213/49	50/41	79/102	7.5
GCMT	219/35	52/38	93/86	7.5
This study	198/54	46/50	63/115	7.5

Table S5. Results of the subevent analysis

#	Delayed time (s)	Longitude (°)	Latitude (°)	Depth (km)	M_w	Strike (°)	Dip (°)	Rake (°)
1	23.0	136.5903	37.8026	4.78	7.26	148.3	47.8	84.2
2	14.0	137.3717	37.5385	18.39	7.18	93.8	42.9	143.5
3	8.0	137.1151	37.4523	12.34	7.19	220.0	63.5	113.3
4	27.5	136.8614	37.1989	18.39	7.14	224.5	81.6	94.0
5	23.0	137.3141	37.5108	17.64	7.16	118.7	40.3	174.0

Data S1: decompose3d: 3D displacements from SAR pixel offset analysis

Data S2: fault_model_v6s: Fault model obtained by inverting SAR pixel offset and GNSS displacements

Data S3: disp_fault_model_v6_v6s: Surface displacements calculated by the fault model (fault_model_v6s)

Data S4: POT_filtered: Subsampled range and azimuth displacement data obtained from pixel offset analysis

Data S5: GNSS: GNSS displacement data prepared using GEONET F5 solution

Data S6: Hypocenters.dat: Hypocenters of the aftershocks of the 2024 Noto Peninsula earthquake

Data S7: CoastU3d_v03.csv: 3D displacements along the northern coast of Noto Peninsula, extracted from decompose3d

Data S8: Coastal uplift measurement.xlsx: Uplift data measured in the field

REFERENCES AND NOTES

1. J.-P. Avouac, Mountain building, erosion, and the seismic cycle in the Nepal Himalaya. *Adv. Geophys.* **46**, 1–80 (2003).

2. S. J. Dadson, N. Hovius, H. Chen, W. B. Dade, M.-L. Hsieh, S. D. Willett, J.-C. Hu, M.-J. Horng, M.-C. Chen, C. P. Stark, D. Lague, J.-C. Lin, Links between erosion, runoff variability and seismicity in the Taiwan orogen. *Nature* **426**, 648–651 (2003).

3. O. Marc, R. Behling, C. Andermann, J. M. Turowski, L. Illien, S. Roessner, N. Hovius, Long-term erosion of the Nepal Himalayas by bedrock landsliding: The role of monsoons, earthquakes and giant landslides. *Earth Surf. Dyn.* **7**, 107–128 (2019).

4. T. Yoshikawa, S. Kaizuka, Y. Ôta, Mode of crustal movement in the Late Quaternary on the southeast coast of Shikoku Southwestern Japan. *Geogr. Rev. Jpn.* **37**, 627–648 (1964).

5. Y. Ota, K. Hirakawa, Marine terraces and their deformation in Noto Peninsula, Japan Sea side of central Japan. *Geogr. Rev. Jpn.* **52**, 169–189 (1979).

6. H. Sato, The relationship between Late Cenozoic tectonic events and stress field and basin development in northeast Japan. *J. Geophys. Res. Solid Earth* **99**, 22261–22274 (1994).

7. Y. Okamura, M. Watanabe, R. Morijiri, M. Satoh, Rifting and basin inversion in the eastern margin of the Japan Sea. *Island Arc* **4**, 166–181 (1995).

8. T. Uchide, T. Shiina, K. Imanishi, Stress map of Japan: Detailed nationwide crustal stress field inferred from focal mechanism solutions of numerous microearthquakes. *J. Geophys. Res. Solid Earth* **127**, (2022).

9. Y. Ota, T. Matsuda, K. Hirakawa, Active faults in Noto Peninsula, Central Japan. *Quat. Res. Daiyonki Kenkyu* **15**, 109–128 (1976).

10. T. Nishimura, Y. Hiramatsu, Y. Ohta, Episodic transient deformation revealed by the analysis of multiple GNSS networks in the Noto Peninsula, central Japan. *Sci. Rep.* **13**, 8381 (2023).

11. K. Yoshida, M. Uno, T. Matsuzawa, Y. Yukutake, Y. Mukuhira, H. Sato, T. Yoshida, Upward earthquake swarm migration in the northeastern Noto peninsula, japan, initiated from a deep ring-shaped cluster: Possibility of fluid leakage from a hidden magma system. *J. Geophys. Res. Solid Earth* **128**, e2022JB026047 (2023).

12. R. Michel, J.-P. Avouac, J. Taboury, Measuring ground displacements from SAR amplitude images: Application to the Landers Earthquake. *Geophys. Res. Lett.* **26**, 875–878 (1999).

13. D. Petley, Landslides from the 1 January 2024 Noto Peninsula Earthquake in Japan (2024). https://eos.org/thelandslideblog/noto-peninsula-earthquake-1.

14. Geospatial Information Authority of Japan, Information on the 2024 Noto Peninsula earthquake (in Japanese) (2024). www.gsi.go.jp/BOUSAI/20240101_noto_earthquake.html.

15. Ministry of Land, Infrastructure, Transport and Tourism, Investigation for large earthquakes occurring in the Sea of Japan (2014). www.mlit.go.jp/river/shinngikai_blog/daikibojishinchousa/.

16. H. Horikawa, Characterization of the 2007 Noto Hanto, Japan, earthquake. *Earth Planets Space* **60**, 1017–1022 (2008).

17. S. Yoshida, T. Noguchi, T. Cho, M. Shimazaki, M. Koba, H. Sato, Offshore crustal movement associated with the Noto Hanto earthquake. *Bull. Earthq. Res. Inst. Univ. Tokyo* **82**, 333–344 (2007).

18. K. Asano, T. Iwata, Source-rupture process of the 2007 Noto Hanto, Japan, earthquake estimated by the joint inversion of strong motion and GPS data. *Bull. Seismol. Soc. Am.* **101**, 2467–2480 (2011).

19. T. Yamada, K. Mochizuki, M. Shinohara, T. Kanazawa, A. Kuwano, K. Nakahigashi, R. Hino, K. Uehira, T. Yagi, N. Takeda, S. Hashimoto, Aftershock observation of the Noto Hanto earthquake in 2007 using ocean bottom seismometers. *Earth Planets Space* **60**, 1005–1010 (2008).

20. S. Sakai, A. Kato, T. Iidaka, T. Iwasaki, E. Kurashimo, T. Igarashi, N. Hirata, T. Kanazawa, Highly resolved distribution of aftershocks of the 2007 Noto Hanto Earthquake by a dense seismic observation. *Earth Planets Space* **60**, 83–88 (2008).

21. Y. Fukushima, T. Ozawa, M. Hashimoto, Fault model of the 2007 Noto Hanto earthquake estimated from PALSAR radar interferometry and GPS data. *Earth Planets Space* **60**, 99–104 (2008).

22. Y. Hiramatsu, K. Moriya, T. Kamiya, M. Kato, T. Nishimura, Fault model of the 2007 Noto Hanto earthquake estimated from coseismic deformation obtained by the distribution of littoral organisms and GPS: Implication for neotectonics in the northwestern Noto Peninsula. *Earth Planets Space* **60**, 903–913 (2008).

23. H. Katagawa, M. Hamada, S. Yoshida, H. Kadosawa, A. Mitsuhashi, Y. Kono, Y. Kinugasa, Geological development of the west sea area of the Noto peninsula district in the neogene tertiary to quaternary, central Japan. *J. Geogr.* **114**, 791–810 (2005).

24. R. Okuwaki, Y. Yagi, A. Murakami, Y. Fukahata, A multiplex rupture sequence under complex fault network due to preceding earthquake swarms during the 2024 MW 7.5 Noto Peninsula, Japan, earthquake. *Geophys. Res. Lett.* **51**, e2024GL109224 (2024).

25. M. Shishikura, T. Echigo, Y. Namegaya, Activity of the off-shore active faults along the northern coast of the Noto Peninsula deduced from the height distribution of the lower marine terrace and emerged sessile assemblage. *Active Fault Res.* **2020**, 33–49 (2020).

26. M. Hamada, Y. Hiramatsu, M. Oda, H. Yamaguchi, Fossil tubeworms link coastal uplift of the northern Noto Peninsula to rupture of the Wajima-oki fault in AD 1729. *Tectonophysics* **670**, 38–47 (2016).

27. M. Shishikura, T. Echigo, Y. Namegaya, Evidence for coseismic and aseismic uplift in the last 1000 years in the focal area of a shallow thrust earthquake on the Noto Peninsula, west-central Japan. *Geophys. Res. Lett.* **36**, L02307 (2009).

28. M. Shishikura, T. Echigo, Y. Namegaya, R. Tateishi, R. Goto, H. Maemoku, Coastal emergence and formation of marine terrace associated with coseismic uplift during the 2024 Noto Peninsula Earthquakes. *Quatern. Res.* **63**, 169–174 (2024).

29. R. W. Gallois, A recent landslide on the east Devon coast, UK. *Q. J. Eng. Geol. Hydrogeol.* **40**, 29–34 (2007).

30. S. Aksay, S. Ivy-Ochs, K. Hippe, L. Grämiger, C. Vockenhuber, Slope failure in a period of increased landslide activity: Sennwald rock avalanche. *Switzerland. Geosci. J.* **11**, 331 (2021).

31. S. Zhang, R. Li, F. Wang, A. Iio, Characteristics of landslides triggered by the 2018 Hokkaido Eastern Iburi earthquake, Northern Japan. *Landslides* **16**, 1691–1708 (2019).

32. R. J. Chandler, The Magheramorne Landslide, Northern Ireland: Mechanics and remedial measures. *J. Jpn. Landslide Soc.* **38**, 327–333 (2002).

33. M. Van Den Eeckhaut, T. Vanwalleghem, J. Poesen, G. Govers, G. Verstraeten, L. Vandekerckhove, Prediction of landslide susceptibility using rare events logistic regression: A case-study in the Flemish Ardennes (Belgium). *Geomorphology* **76**, 392–410 (2006).

34. O. Hungr, S. Leroueil, L. Picarelli, The Varnes classification of landslide types, an update. *Landslides* **11**, 167–194 (2014).

35. S. Fujiwara, H. Yarai, T. Kobayashi, Y. Morishita, T. Nakano, B. Miyahara, H. Nakai, Y. Miura, H. Ueshiba, Y. Kakiage, H. Une, Small-displacement linear surface ruptures of the 2016 Kumamoto earthquake sequence detected by ALOS-2 SAR interferometry. *Earth Planets Space* **68**, 1–17 (2016).

36. Y. Fukushima, D. Ishimura, Characteristics of secondary-ruptured faults in the Aso Caldera triggered by the 2016 Mw 7.0 Kumamoto earthquake. *Earth Planets Space* **72**, 175 (2020).

37. X. Xu, D. T. Sandwell, L. A. Ward, C. W. D. Milliner, B. R. Smith-Konter, P. Fang, Y. Bock, Surface deformation associated with fractures near the 2019 Ridgecrest earthquake sequence. *Science* **370**, 605–608 (2020).

38. H. Z. Yin, X. Xu, J. S. Haase, R. Douilly, D. T. Sandwell, B. M. de Lepinay, Surface deformation surrounding the 2021 Mw 7.2 Haiti earthquake illuminated by InSAR observations. *Bull. Seismol. Soc. Am.* **113**, 41–57 (2022).

39. Earthquake Research Committee, Long-term evaluation of the offshore active faults on the Japan Sea side: north of Hyogo prefecture to Joetsu region of Niigata prefecture (version August 2024, in Japanese) (2024). www.jishin.go.jp/main/chousa/24aug_sea_of_japan/sea_of_japan_honbun.pdf.

40. F. Waldhauser, W. L. Ellsworth, A double-difference earthquake location algorithm: Method and application to the Northern Hayward Fault, California. *Bull. Seismol. Soc. Am.* **90**, 1353–1368 (2000).

41. D. R. Shelly, D. P. Hill, F. Massin, J. Farrell, R. B. Smith, T. Taira, A fluid-driven earthquake swarm on the margin of the Yellowstone caldera. *J. Geophys. Res. Solid Earth* **118**, 4872–4886 (2013).

42. H. Ueno, S. Hatakeyama, T. Aketagawa, J. Funasaki, N. Hamada, Improvement of hypocenter determination procedures in the Japan Meteorological Agency (in Japanese with English abstract). *Q. J. Seismol.* **65**, 123 (2002).

43. National Research Institute for Earth Science and Disaster Resilience, NIED Hi-net (2019). https://doi.org/10.17598/nied.0003.

44. D. Massonnet, M. Rossi, C. Carmona, F. Adragna, G. Peltzer, K. Feigl, T. Rabaute, The displacement field of the Landers earthquake mapped by radar interferometry. *Nature* **364**, 138–142 (1993).

45. T. Ozawa, E. Fujita, H. Ueda, Crustal deformation associated with the 2016 Kumamoto Earthquake and its effect on the magma system of Aso volcano. *Earth Planets Space* **68**, 186 (2016).

46. C. Liang, E. J. Fielding, Interferometry with ALOS-2 full-aperture ScanSAR data. *IEEE Trans. Geosci. Remote Sens.* **55**, 2739–2750 (2017).

47. Y. Morishita, T. Kobayashi, Three-dimensional deformation and its uncertainty derived by integrating multiple SAR data analysis methods. *Earth Planets Space* **74**, 16 (2022).

48. T. Yamashita, Y. Morishita, T. Kobayashi, Mitigation of ionospheric noise in azimuth offset based on the split-spectrum method. *IEEE Trans. Geosci. Remote Sens.* **60**, 10.1109/TGRS.2021.3073511 (2022).

49. D. Raucoules, M. de Michele, Assessing ionospheric influence on L-band SAR data: Implications on coseismic displacement measurements of the 2008 Sichuan Earthquake. *IEEE Geosci. Remote Sens. Lett.* **7**, 286–290 (2010).

50. U. Wegmüller, C. Werner, T. Strozzi, A. Wiesmann, "Ionospheric electron concentration effects on SAR and INSAR" in *IEEE International Symposium on Geoscience and Remote Sensing* (IEEE, 2006), pp. 3731–3734.

51. T. Kobayashi, Y. Takada, M. Furuya, M. Murakami, Locations and types of ruptures involved in the 2008 Sichuan earthquake inferred from SAR image matching. *Geophys. Res. Lett.* **36**, L07302 (2009).

52. Geospatial Information Authority of Japan, Crustal deformation associated with the 5 May 2023 earthquake in the Noto region, Ishikawa Prefecture, using SAR interferometric analysis of the ALOS-2 observation data (in Japanese) (2023). www.gsi.go.jp/uchusokuchi/20230505noto.html.

53. N. Takamatsu, H. Muramatsu, S. Abe, Y. Hatanaka, T. Furuya, Y. Kakiage, K. Ohashi, C. Kato, K. Ohno, S. Kawamoto, New GEONET analysis strategy at GSI: Daily coordinates of over 1300 GNSS CORS in Japan throughout the last quarter century. *Earth Planets Space* **75**, 49 (2023).

54. T. J. Wright, B. E. Parsons, Z. Lu, Toward mapping surface deformation in three dimensions using InSAR. *Geophys. Res. Lett.* **31**, L01607 (2004).

55. S. Leprince, S. Barbot, F. Ayoub, J.-P. Avouac, Automatic and precise orthorectification, coregistration, and subpixel correlation of satellite images, application to ground deformation measurements. *IEEE Trans. Geosci. Remote Sens.* **45**, 1529–1558 (2007).

56. COSI-Corr: Measuring Ground Deformation from Optical Satellite and Aerial images. www.tectonics.caltech.edu/slip_history/spot_coseis/download_software.html.

57. T. Inoue, Y. Okamura, 1:200,000 Marine geological map around the northern part of Noto Peninsula with explanatory notes (Geological Survey of Japan, AIST, 2010).

58. M. Nikkhoo, T. R. Walter, Triangular dislocation: An analytical, artefact-free solution. *Geophys. J. Int.* **201**, 1119–1141 (2015).

59. M. Kikuchi, H. Kanamori, Inversion of complex body waves—III. *Bull. Seismol. Soc. Am.* **81**, 2335–2350 (1991).

60. Z. Jia, Z. Jin, M. Marchandon, T. Ulrich, A.-A. Gabriel, W. Fan, P. Shearer, X. Zou, J. Rekoske, F. Bulut, A. Garagon, Y. Fialko, The complex dynamics of the 2023 Kahramanmaraş, Turkey, M_w 7.8-7.7 earthquake doublet. *Science* **381**, 985–990 (2023).

61. National Research Institute for Earth Science and Disaster Resilience, NIED KiK-net (2019). https://doi.org/10.17598/nied.0004.

62. L. Zhu, L. A. Rivera, A note on the dynamic and static displacements from a point source in multilayered media. *Geophys. J. Int.* **148**, 619–627 (2002).

63. Japan Meteorological Agency, Tidal and sea level data. www.data.jma.go.jp/kaiyou/db/tide/suisan/index.php.

64. K.-Y. Kim, Vertical distribution and seasonality of intertidal macroalgae on the coast of Hawon-Pando, Southwestern Korea. *J. Korean soc. Oceanog.* **34**, 172–178 (1999).

65. T. Nakata, T. Imaizumi, *Digital Active Fault Map of Japan* (University of Tokyo Press, 2002).

66. T. Yoshikawa, K. Kano, Y. Yanagisawa, M. Komazawa, M. Joshima, E. Kikawa, Geology Map of Suzumisaki, Noto-iida, and Horyuzan (Geological Survey of Japan, AIST, 2002); www.gsj.jp/Map/JP/docs/5man_doc/10/10_003.htm.

67. National Research Institute for Earth Science and Disaster Resilience, Digital archive for landslide distribution maps (2014). https://dil-opac.bosai.go.jp/publication/nied_tech_note/landslidemap/index.html.

68. Japan Geospatial Information Center. www.geospatial.jp/ckan/dataset/2024-notowest-ground.
