# Supplementary material for: Landscape changes caused by the 2024 Noto Peninsula earthquake in Japan
Source: Sci Adv. 2024 Dec 4;10(49):eadp9193. doi: 10.1126/sciadv.adp9193 (PMC11616706; doi:10.1126/sciadv.adp9193)
Supplement: Supplementary file 2 — Data S1 to S8 [file sciadv.adp9193_data_s1_to_s8.zip › adp9193_data_s1.zip!/decompose3d/3Ddispl_v03.pdf]

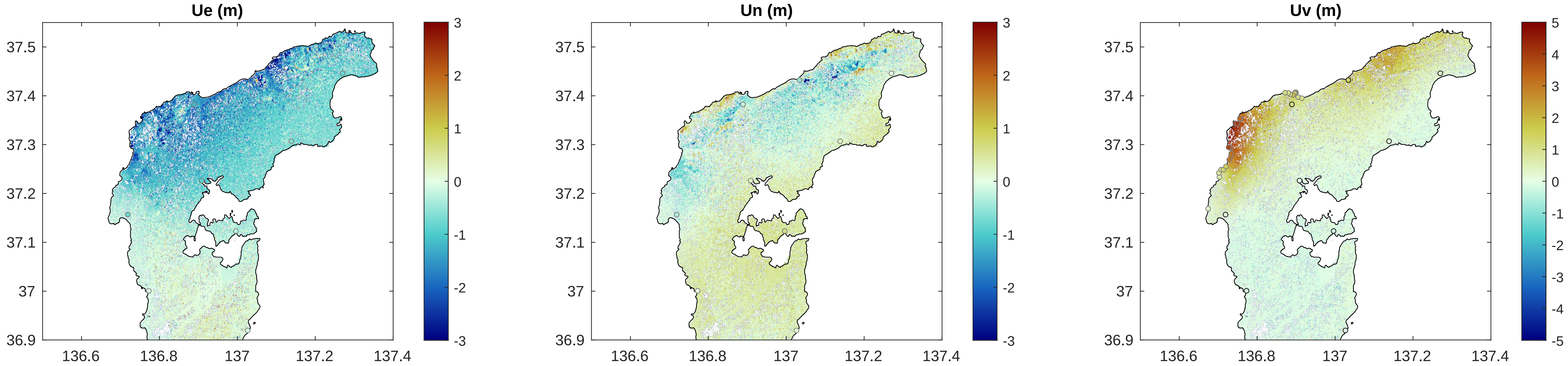
Ue (m)
Un (m)
Uv (m)
37.5
37.4
37.3
37.2
37.1
37
36.9
136.6
136.8
137
137.2
137.4
3
2
1
0
-1
-2
-3
5
4
3
2
1
0
-1
-2
-3
-4
-5
